# Upregulation of SNTB1 correlates with poor prognosis and promotes cell growth by negative regulating PKN2 in colorectal cancer

**DOI:** 10.1186/s12935-021-02246-7

**Published:** 2021-10-18

**Authors:** Liya Liu, Youqin Chen, Xiaoying Lin, Meizhu Wu, Jiapeng Li, Qiurong Xie, Thomas J. Sferra, Yuying Han, Huixin Liu, Liujing Cao, Mengying Yao, Jun Peng, Aling Shen

**Affiliations:** 1 Academy of Integrative Medicine, Fujian University of Traditional Chinese Medicine, 1 Qiuyang Road, Minhou Shangjie, Fuzhou, 350122 Fujian China; 2 Fujian Key Laboratory of Integrative Medicine on Geriatrics, Fujian University of Traditional Chinese Medicine, 1 Qiuyang Road, Minhou Shangjie, Fuzhou, 350122 Fujian China; 3 Department of Pediatrics, Case Western Reserve University School of Medicine, UH Rainbow Babies and Children’s Hospital, Cleveland, OH 44106 USA; 4 Department of Physical Education, Fujian University of Traditional Chinese Medicine, Fuzhou, 350122 Fujian China

**Keywords:** Colorectal cancer, Syntrophin beta 1, Growth, Overall survival, Protein kinase N2

## Abstract

**Background:**

Colorectal cancer (CRC) is one of the most highly malignant tumors and has a complicated pathogenesis. A preliminary study identified syntrophin beta 1 (SNTB1) as a potential oncogene in CRC. However, the clinical significance, biological function, and underlying mechanisms of SNTB1 in CRC remain largely unknown. Thus, the present study aimed to investigate the role of SNTB1 in CRC.

**Methods:**

The expression profile of SNTB1 in CRC samples was evaluated by database analysis, cDNA array, tissue microarray, quantitative real-time PCR (qPCR), and immunohistochemistry. SNTB1 expression in human CRC cells was silenced using short hairpin RNAs (shRNA)/small interfering RNAs (siRNA) and its mRNA and protein levels were assessed by qPCR and/or western blotting. Cell viability, survival, cell cycle, and apoptosis were determined by the CCK-8 assay, colony formation, and flow cytometry assays, respectively. A xenograft nude mouse model of CRC was established to validate the roles of SNTB1 in vivo. Immunohistochemistry and TUNEL staining were used to determine the expression of SNTB1, PCNA, and cell apoptosis in tissue samples. Isobaric tag for relative and absolute quantification (iTRAQ) was used to analyze the differentially expressed proteins after knockdown of SNTB1 in CRC cells. Silence of protein kinase N2 (PKN2) using si-PNK2 was performed for rescue experiments.

**Results:**

SNTB1 expression was increased in CRC tissues compared with adjacent noncancerous tissues and the increased SNTB1 expression was associated with shorter overall survival of CRC patients. Silencing of SNTB1 suppressed cell viability and survival, induced cell cycle arrest and apoptosis in vitro, and inhibited the growth of CRC cells in vivo. Further elucidation of the regulation of STNB1 on CRC growth by iTRAQ analysis identified 210 up-regulated and 55 down-regulated proteins in CRC cells after SNTB knockdown. A PPI network analysis identified PKN2 as a hub protein and was up-regulated in CRC cells after SNTB1 knockdown. Western-blot analysis further confirmed that SNTB1 knockdown significantly up-regulated PKN2 protein expression in CRC cells and decreased the phosphorylation of both ERK1/2 and AKT. Moreover, rescue experiments indicated that PKN2 knockdown significantly rescued SNTB1 knockdown-mediated decrease in cell viability, survival, and increase of cell cycle arrest at G0/G1 phase and apoptosis of CRC cells.

**Conclusions:**

These findings indicate that SNTB1 is overexpressed in CRC. Elevated SNTB1 levels are correlated with shorter patient survival. Importantly, SNTB1 promotes tumor growth and progression of CRC, possibly by reducing the expression of PKN2 and activating the ERK and AKT signaling pathway. Our study highlights the potential of SNTB1 as a new prognostic factor and therapeutic target for CRC.

**Supplementary Information:**

The online version contains supplementary material available at 10.1186/s12935-021-02246-7.

## Background

Colorectal cancer (CRC) is the third most frequently diagnosed cancer (6.1% of all cancers) and the second leading cause of cancer death (9.2% of all cancer deaths) [1]. Although great strides have been made in the treatment of CRC, metastasis, recurrence, and chemoresistance remain major obstacles to delivering effective treatment in all patients [2–4]. Therefore, obtaining a better understanding of the molecular mechanisms responsible for the rapid growth and tumorigenic properties of CRC may lead to novel therapeutic strategies for this disease.

In preliminary published work, using a gene expression profile microarray to screen for differential expressed genes (DEGs) between paired CRC and noncancerous tissues [5], we found the level of syntrophin beta 1 (SNTB1) to be upregulated in CRC tissue (GEO ID: GSE113513). However, the clinical significance, biological function, and underlying regulating mechanisms of SNTB1 in most cancers, including CRC, remain largely unknown. This encouraged us to further explore the role of SNTB1 in CRC.

SNTB1 is a member of syntrophin gene family which consists of five homologous isoforms, α1, β1, β2, γ1 and γ2 [6–8]. Two pleckstrin homology (PH) domains, a PDZ domain, and a conserved syntrophin unique (SU) region constitute the structure of SNTB1 and are sites of interaction with other proteins [9]. SNTB1 is associated with dystrophin and dystrophin-related proteins, which is one of the members of syntrophin gene family and mainly expressed in skeletal and smooth muscle, liver and kidney and expressed at low levels in many other tissues, including colorectal tissue [6]. Loss or reduction of SNTB1 is closely associated with Duchenne muscular dystrophy and Becker’s muscular dystrophy [7]. Recent studies revealed that the functional variants of SNTB1 correlated with several diseases, including acute pancreatitis, oral cancer, lung cancer, and severe myopia [8, 10–12]. Moreover, lower SNTB1 expression correlated with shorter survival rate in lung adenocarcinoma patients [12], while SNTB1 over-expression associated with shorter survival of CRC patients [13]. However, the clinical significance, biological function and underlying mechanism of SNTB1 in CRC remain largely unknown.

In the present study, bioinformatics analysis of online databases, cDNA array, and tissue microarray (TMA) were used to evaluate the expression profile of SNTB1 and the relationship between SNTB1 expression and clinical pathological parameters in CRC. Moreover, a series of in vitro and in vivo experiments using representative CRC cell lines and xenograft nude mice models were conducted to examine the functional role of SNTB1. Furthermore, the underlying mechanisms of SNTB1 on tumor growth were explored and rescue experiments were performed to explore the regulatory effects.

## Methods

### Materials and reagents

The antibodies against SNTB1 (GTX46866) and PCNA (GTX100539) were purchased from GeneTex (San Antonio, TX, USA). The antibodies against Caspase-3 (9662), Caspase-9 (9508), p-ERK1/2 (9102), ERK1/2 (4370), AKT (4691), p-AKT (4060), p-cJun (3270), p-p38 MAPK(9216), p38 MAPK(8690), and GAPDH (2118) were purchased from Cell Signaling Technology (Danvers, MA, USA). The antibody against SNTB1 (PA5-55143), Fetal Bovine Serum (FBS), Trypsin-EDTA (0.25%), Pierce TM BCA Protein Assay kit, and FxCycle PI/RNase Staining Solution were purchased from Thermo Fisher Scientific (Carlsbad, CA, USA). The antibody against PKN2 (14608-1-AP) was purchased from Proteintech Group (Wuhan, Hubei, China). Annexin-V-AbFluor™ 647 Apoptosis Detection kit and CCK8 kit were obtained from Abbkine (Wuhan, Hubei, China).

### Differential expressed genes (DEGs) analysis

In prior work, we screened DEGs on 14 pairs of CRC primary lesions and surrounding non-cancerous tissues (GEO Submission: GSE113513) [5]. Among these DEGs, 8 DEGs including SNTB1 were selected for further investigation due to the limited amount of published data regarding their involvement in carcinogenesis. In this study, mRNA expression of SNTB1in CRC and control tissues was analyzed thorough TCGA (https://cancergenome.nih.gov/) [14].

### Cell lines and culture

Both human CRC cell lines HCT116 and RKO were obtained from the Cell Bank of the Chinese Academy of Sciences (Shanghai, China). HCT116 cells were cultured in M5′A medium (KeyGEN, Jiangsu, China) and RKO cells were cultured in MEM-alpha medium (Thermo Fisher Scientific) supplemented with 10% FBS (Thermo Fisher Scientific) and 1% penicillin-streptomycin (Hyclone, Logan, UT, USA). All cells were cultured at 37 °C in a humidified atmosphere with 5% CO_2_. Short tandem repeat genotyping by qPCR was performed to monitor for mycoplasma contamination of cells.

### Lentiviral transduction and high-content screening for cell growth

High-content screening (HCS) was performed to assess the growth of CRC cells. Briefly, control shRNA lentivirus and shRNA lentivirus targeting 8 DEGs (the sequences of shRNAs are listed in Additional file 4: Table S1) were constructed by Shanghai GeneChem (Shanghai, China). HCT116 cells were seeded in 12-well plates for 16 h prior to lentivirus transduction and then transduced by adding the shRNA lentiviral particles (multiplicity of infection: 10) with GFP into the cell culture medium according to the protocol of the manufacturer. At the end of transduction, 2 × 10^3^ cells in 100 µL of complete medium were reseeded into 96-well plates. Cell growth was monitored every day for five days using the Pathway 855 high-content image analysis platform (BD Biosciences, San Jose, CA, USA).

### Cell transfection

Three different siRNAs: anti-SNTB1 (si-SNTB1), anti-PKN2 (si-PKN2), and control siRNA (si-Ctrl) were purchased from Ribobio (Guangzhou, Guangdong, China). Cells were transfected with siRNAs or si-Ctrl at a concentration of 50 nM using Lipofectamine RNAiMax (Thermo Fisher Scientific) according to manufacturer’s instructions for 6–8 h. The cells were then cultured with complete medium at 37 °C in a humidified atmosphere with 5% CO_2_ for indicated time points prior to use for experiments.

### QPCR analysis

The total RNA was isolated from cells using RNAiso Plus reagent (Takara, Beijing, China). Reverse transcription into complementary DNA (cDNA) was amplified according to the manufacturer’s instructions using the PrimeScript RT reagent kit (Takara). Tissue cDNA array containing 79 primary CRC and 15 noncancerous colorectal tissues (Cat#: cDNA-hcola095su01) was purchased from Shanghai Outdo Biotech Company (Shanghai, China) and the levels of mRNA encoding SNTB1 and GAPDH were measured using an ABI 7500 Fast Real-Time PCR System (Applied Biosystems) and the SYBR Premix Ex Tag (Takara). The conditions for qPCR were as follows: pre-denaturation (95 °C for 10 min), denaturation (95 °C for 15 s), and annealing and extension (60 °C for 60 s) for a total 40 cycles. GAPDH was used as an internal control. Primer sequences are shown in Additional file 5: Table S2. mRNA levels are presented as: 2ΔΔCt (with Ct being the cycle threshold), where ΔCt [Ct (target gene) − Ct (GAPDH)]. Clinicopathologic features of CRC patients represented in the cDNA array are summarized in Additional file 6: Table S3.

### Tissue microarray

TMA containing 70 pairs of CRC and noncancerous colorectal tissues were obtained from Shanghai Outdo Biotech Company (Shanghai, China; Cat#: HColA180Su15). IHC was conducted to detect the expression of SNTB1 in CRC samples using an antibody against SNTB1 (Rabbit monoclonal to SNTB1; dilution 1:100; Thermo Fisher Scientific) as described previously [15]. Scoring was carried out using a grading system based on staining intensity (no staining, 0; weak, 1; moderate, 2; strong, 3) and percentage of positive-staining cells (1–25% positive, 1; 26–50%, 2; 51–75%, 3; 76–100%, 4) [16]. The final score was calculated as intensity score × percentage score. Clinicopathological features of CRC patients are summarized in Additional file 7: Table S4.

### Western-blot analysis

Cells were harvested and lysed in RIPA lysis buffer (Beyotime, Jiangsu, China) containing 1 mM phenylmethylsulfonyl fluoride (PMSF) and protease inhibitors. The Pierce BCA Protein Assay Kit (Thermo Fisher Scientific) was used to measure the concentrations of total protein. Equal amount of total protein lysate was separated on 10% SDS-polyacrylamide gel and transferred to PVDF membranes (Millipore, Bedford, MA, USA). Next the membranes were blocked with 5% skim milk in TBST at room temperature for 2 h, incubated overnight at 4 ℃ with primary antibodies (all 1:1000), and followed by incubation with a horseradish peroxidase (HRP)-conjugated goat anti-rabbit secondary antibody (1:2000). Proteins were visualized using an ECL imager (Thermo Fisher Scientific, USA) and band intensities were quantified using ImageLab software. The expression of GAPDH was used as a control. Three independent experiments were performed for each assay.

### CCK-8 assay

Transduced cells were re-seeded into 96-well plates (2000 cells per well) and cultured at 37 °C and 5% CO_2_ for the indicated time points. Cell Counting Kit-8 reagent (10 uL; Abbkine, Wuhan, Hubei, China) was added to each well, plates were incubated for an additional 2 h at 37 °C, and the optical density (OD) was measured at a wavelength of 450 nm. The cell viability was calculated based on the OD for each group.

### Colony formation assay

Transduced cells were seeded into 12-well plates at a density of 500 cells per well and cultured at 37 °C and 5% CO_2_ for 10-14 days. Cells were fixed in 4% paraformaldehyde for 20 min and stained with 0.1% crystal violet (Solarbio, Beijing, China) for 20 min at room temperature. Colonies were manually counted. Each assay was performed in triplicate.

### Cell cycle and apoptosis analysis

For the cell cycle assay, transduced cells were collected and fixed with 70% ethanol at 4 °C overnight. The fixed cells were centrifuged at 2000 rpm for 3 min and washed, followed by incubation with a mixture of FxCycle PI/RNase Staining Solution (Thermo Fisher Scientific) for 30 min at room temperature. FACS (Fluorescence activated Cell Sorting; Becton Dickinson, CA, USA) was used to analyze cell cycle progression using ModfitLT version 3.0 (Verity Software House). For the apoptosis assay, transduced cells were washed twice with ice-cold PBS and incubated with Annexin-V-AbFlour^TM^ 647 Apoptosis Detection Kit solution (Abbkine, Wuhan, Hubei, China). The apoptotic rate was analyzed using FACS.

### In vivo experiments

A xenograft nude mouse model was constructed to investigate the effect of SNTB1 knockdown on tumor growth. Male BALB/c nude mice (6–8 weeks old) were obtained from Shanghai Laboratory Animal Center at the Chinese Academy of Sciences (Shanghai, China) and raised in a specific pathogen-free facility at Fujian University of Traditional Chinese Medicine (Fuzhou, Fujian, China). All animal procedures were approved by the Committee of Fujian University of Traditional Chinese Medicine (Approval No: FJTCM IACUC 2019050). HCT116 cells or RKO cells were transduced with a lentivirus encoding anti-SNTB1 shRNA (sh-SNTB1) or control shRNA (sh-Ctrl). Cells (1 × 10^6^ in 100 µL PBS containing 50% of Matrigel) were injected subcutaneously into the flanks of the nude mice (n = 6). Tumor volume was determined every other day using measurements obtained with a vernier caliper and the following formula: 1/2 (larger diameter × smaller diameter^2^). At the end of the experiment, mice were anesthetized with isoflurane. The IVIS Spectrum live-animal imaging system (PerkinElmer; Santa Clara, CA, USA) was used to capture tumor images. Signal intensity was quantified as the number of photons within the region of interest per second. Mice were sacrificed and tissue collected for use in additional experiments.

### Immunohistochemistry

Tissues were fixed with 4% paraformaldehyde at 4 °C overnight, embedded with paraffin, cut into 5 μm-thick sections, and mounted onto slides. The slides were dehydrated by graded ethanol. Antigens were retrieved by microwave heating for 20 min in sodium citrate-hydrochloric acid buffer. Tissue sections were incubated with an antibody against SNTB1 (1:100; Thermo fisher Scientific) or PCNA (1:800; Genetex). Background staining was assessed by omitting the primary antibody. The intensity of staining was evaluated using a scoring system described in detail in “Tissue microarray” section. The overall staining score was calculated by multiplying the intensity score and percentage score.

### TUNEL assay

Apoptotic cells in tissue sections were detected using terminal deoxynucleotidyl transferase dUTPnick end labeling (TUNEL) according to the manufacturer’s instructions. The percentage of TUNEL-positive cells and staining intensity were evaluated using a scoring system described in detail in “Tissue microarray” section.

### iTRAQ analysis and protein identification

iTRAQ was used to identify differentially expressed proteins (DEPs) [17, 18]. SDS-PAGE electrophoresis was first carried out for protein quantification. The protein samples were cysteine-blocked, digested, and labeled for mass spectrometry (MS) analysis. Two-dimensional liquid chromatography-mass spectrometry (2D-LC-MSMS) analysis including reversed-phase chromatographic separation (Agilent Technologies, Santa Clara, CA, USA) and reversed-phase chromatography on a TripleTOF (AB SCIEX, Framingham, MA, USA) was conducted. Proteins were classified as differentially expressed if their expression differed at least 1.5-fold between the two conditions and if the difference was associated with P < 0.05. These proteins were identified using volcano plots and hierarchical clustering plots. To enrich the biological groups and Kyoto Encyclopedia of Genes and Genomes (KEGG) pathway, the identified proteins were submitted to Omicsbean (http://www.omicsbean.cn/) software [19, 20]. Proteins were then grouped based on their KEGG annotations with P < 0.05 [21]. To better understand the protein–protein interactions among the differentially expressed proteins of each group, we constructed protein–protein interaction (PPI) networks through Omicsbean.

### Statistics analysis

Data were analyzed using SPSS 22.0 software. For survival analysis, SNTB1 mRNA expression in CRC tissues from cDNA array was classified into high or low expression groups based on the mean. Kaplan–Meier survival curves were plotted for high- and low-expression groups and the correlation of SNTB1 expression with overall survival of CRC patients was analyzed using log-rank test. The correlation between SNTB1 expression and CRC patients’ survival in dataset of COAD sourcing from TCGA was analyzed through Kaplan-Meier Plotter (http://kmplot.com/). Student’s t-test or Mann-Whitney U was used for comparisons between two groups. One-way ANOVA or Kruskal–Walis H was applied to assess multiple group comparisons. All quantitative data are presented as the mean ± SD. P < 0.05 (two sided) was considered statistically significant. All experiments were repeated at least three times.

## Results

### SNTB1 is highly expressed in CRC tissues and is associated with poor prognosis

**Fig. 1:**
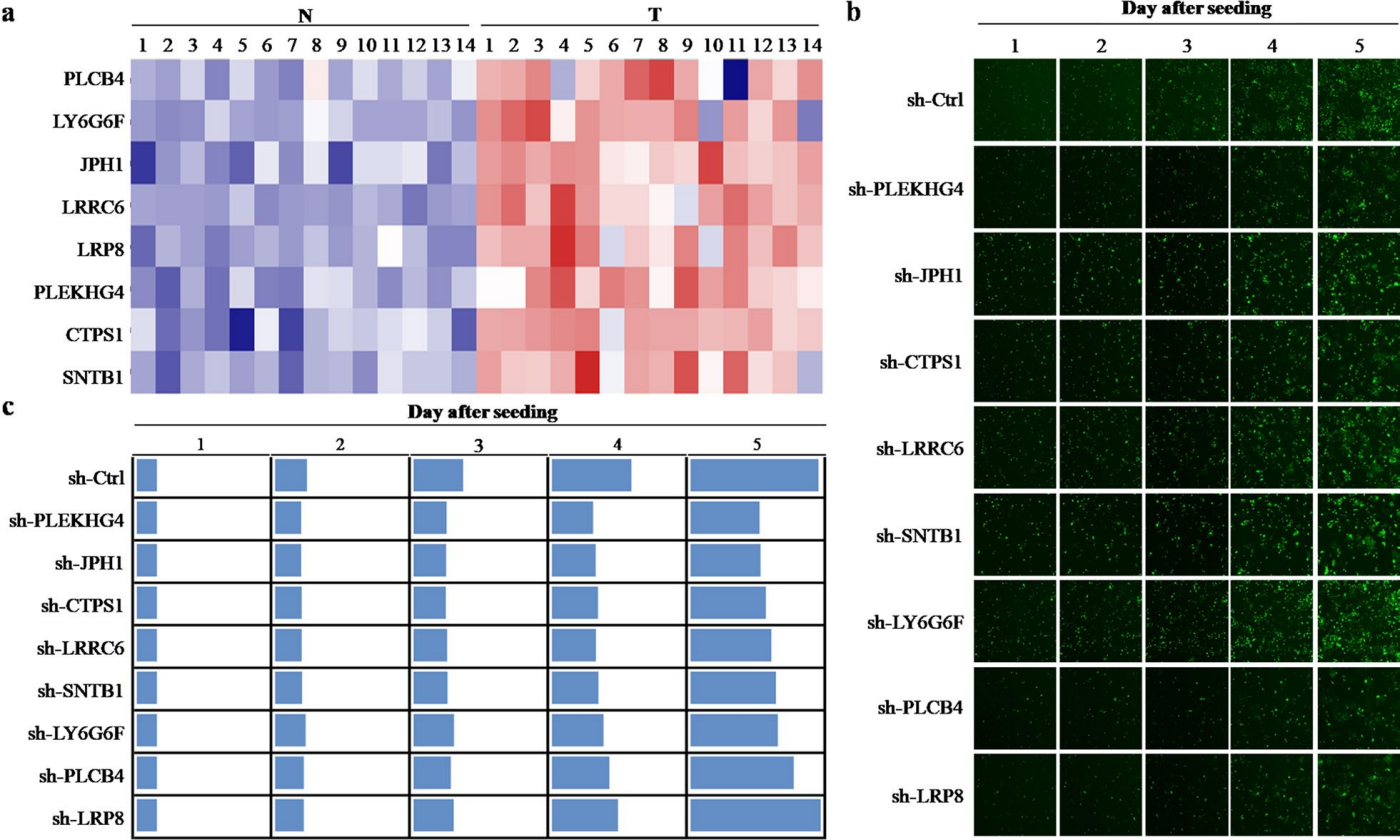
Gene expression profiling and high content screening suggest carcinogenic potential of SNTB1 in CRC. **a** Differentially expressed genes (DEGs) were analyzed from our previous microarray analysis between 14 pairs of colorectal cancer (CRC) tissues (T) and adjacent normal tissues (N). Heatmap of the 8 selected DEGs mRNA expression levels are presented. Blue color indicates low expression and red color high expression. **b** HCT116 cells were transduced with lentivirus encoding shRNAs against these 8 DEGs (SNTB1, PLEKHG4, JPH1, CTPS1, LRRC6, LY6G6F, PLCB4 and LRP8), and cell growth was measured using the using the Pathway 855 high-content image analysis platform. The representative images were taken at a magnification of 200×. **c** Heatmap showing the growth of HCT116 cells. Data were normalized to cell number on day 1 and represented as fold change

In a previous experiment to identify potential oncogenes, we evaluated for differential expressed genes (DEGs) within a CRC microarray containing 14 pairs of CRC tissue and the noncancerous surrounding tissue (GEO ID: GSE113513). Among the identified DEGs, we focused on 8 up-regulated genes (SNTB1, PLEKHG4, JPH1, CTPS1, LRRC6, LY6G6F, PLCB4 and LRP8; Fig. 1a) that had not been extensively studied in CRC. The potential involvements of these genes in CRC were further explored through high-content screening (HCS) using lentivirus delivered shRNAs into HCT116 cells. Seven of the 8 constructs inhibited cell growth (Fig. 1b, c). Considering the largely unexplored role of SNTB1 in oncogenesis [9, 11, 12, 22], we focused on SNTB1 in these studies.

**Fig. 2:**
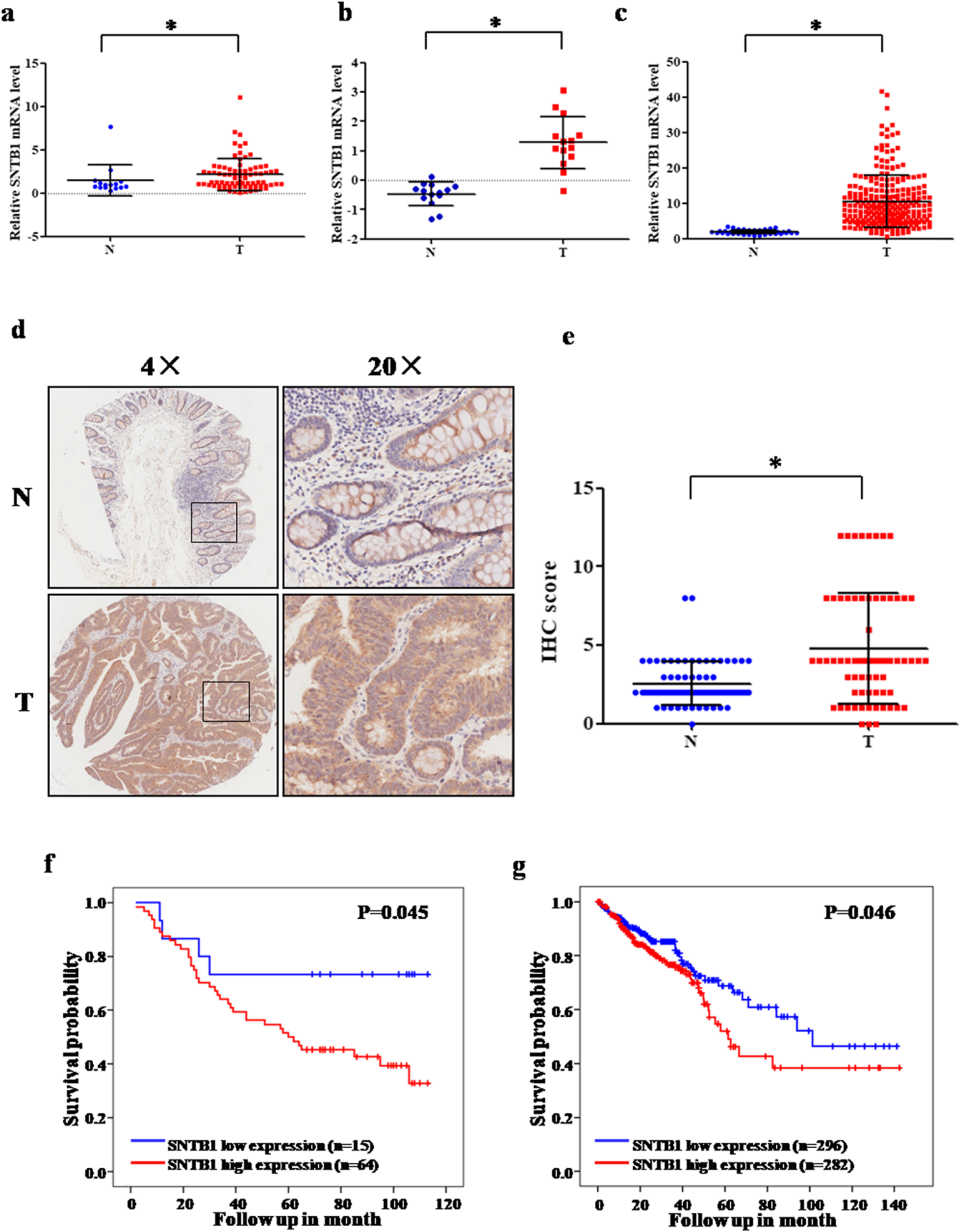
Levels of SNTB1 mRNA and protein in colorectal cancer tissues. **a** SNTB1 mRNA expression in CRC tissues (T: n=79) and non-cancerous colorectal tissues (N: n=15) on the CRC cDNA chip was analyzed by qPCR. GAPDH was used as an internal control. *P < 0.05. **b**, **c** SNTB1 mRNA expression in CRC tissues (T) and non-cancerous colorectal tissues (N) from GEO (**b**) and TCGA (**c**) were analyzed. **d**, **e** SNTB1 protein expression in CRC tissues (T) and non-cancerous colorectal tissues (N) was determined by immunohistochemistry of a tissue microarray. Representative images were taken at magnification of 40× or 200× (right panel); the IHC score was calculated as intensity score × percentage score (left panel, see Methods). *P < 0.05. **f**, **g** Kaplan-Meier plots of survival of CRC patients, stratified by SNTB1 mRNA expression based on cDNA array (**f**) and TCGA database (**g**). Survival was analyzed with log-rank test

QPCR analysis on a cDNA array of 79 primary CRC and 15 noncancerous colorectal tissues indicated that SNTB1 mRNA expression is up-regulated in CRC tissues (Fig. 2a). Similar results were obtained with online data mining using the GEO (GEO ID: GSE113513) and TCGA (Fig. 2b, c) datasets. In a consistent fashion, immunohistochemical (IHC) evaluation of a tissue microarray (TMA) containing 70 pairs of primary CRC lesions and adjacent noncancerous tissues (Fig. 2d, e) confirmed the up-regulation of SNTB1 protein expression in CRC tissues. Correlation analysis of the CRC patients represented in the utilized cNDA array, showed that higher SNTB1 expression (as determined with qPCR-based cDNA array) correlated with shorter overall survival rate (Fig. 2f). There was no correlation between SNTB1 expression and clinicopathological characteristics of CRC patients (Additional file 8: Table S5). Online data mining using the dataset of COAD from TCGA also revealed an association between higher SNTB1 expression and shorter overall survival of CRC patients (Fig. 2g).

### SNTB1 knockdown inhibits cell proliferation and induces cell apoptosis of CRC cells in vitro

To explore the biological function of SNTB1 on CRC cell growth, three different shRNAs specific for SNTB1 were encoded within lentiviruses and used for cell transduction. QPCR and Western-blot analyses revealed significant down-regulation of SNTB1expression on both mRNA (Additional file 1: Figure S1a) and protein (Additional file 1: Figure S1b-c) levels in HCT116 cells with all three shRNAs. Consistently, transfection of 3 different siRNAs against SNTB1 also down-regulated SNTB1 protein expression and attenuated the increase in cell viability of HCT116 cells (Additional file 2: Figure S2a-c). As each of the shRNAs or siRNA had similar effects on SNTB1 expression and cell growth, subsequent experiments were performed with single constructs referred to as “sh-SNTB1”(sh-SNTB1-1) or “si-SNTB1” (si-SNTB1-2).

**Fig. 3:**
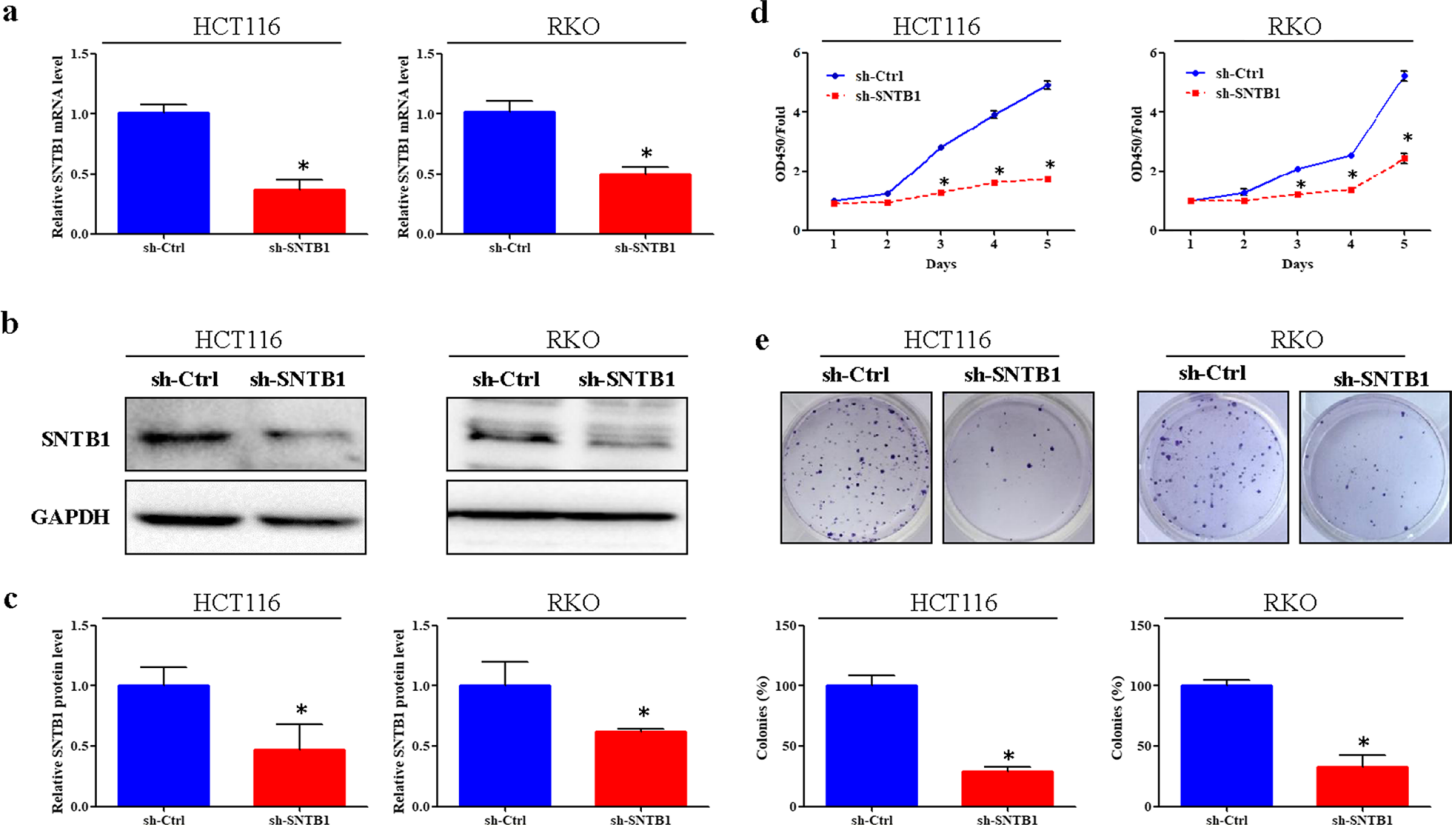
SNTB1 knockdown inhibits colorectal cancer (CRC) cell growth. HCT116 and RKO cells were transduced with lentivirus encoding either anti-SNTB1 small hairpin RNA (sh-SNTB1) or control shRNA (sh-Ctrl). **a **The mRNA levels of SNTB1 were determined by qPCR, GAPDH was used as an internal control. *P < 0.05. **b**, **c** The protein levels of SNTB1 were determined by Western-blot analysis. The representative images of SNTB1 and GAPDH are shown (**b**) and were quantitated using ImageLab software (**c**). GAPDH was used as an internal control and normalized to GAPDH. *P < 0.05. **d** The cell viability of CRC cells was determined by the CCK-8 assay. Data were normalized to the viability on Day 1 and are represented as fold change. *P < 0.05. **e** The colony formation assay was performed to determine cell survival. The images were obtained and the number of colonies formed were determined and normalized to the survival of control cells. *P < 0.05

**Fig. 4:**
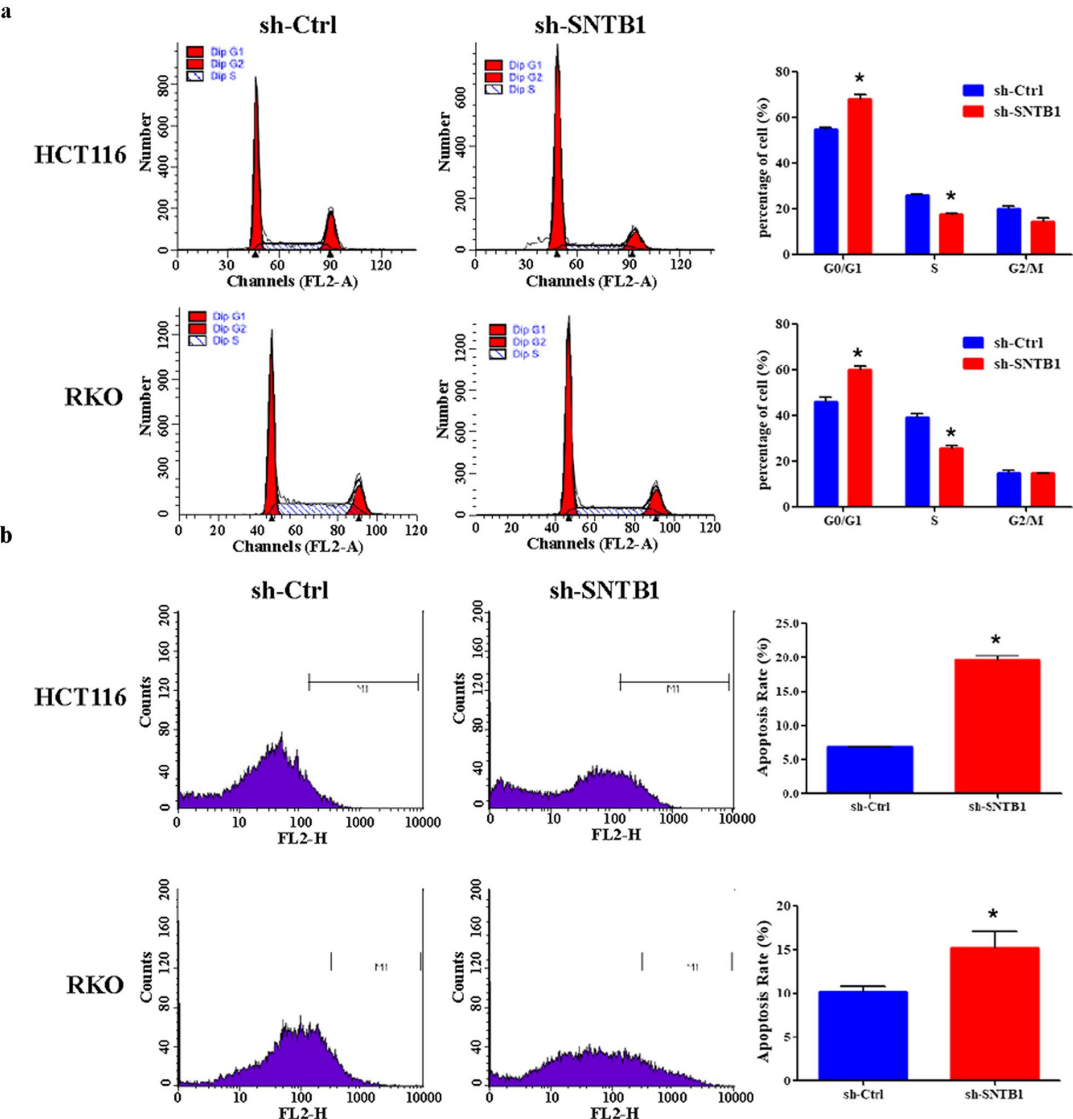
SNTB1 knockdown induces cell cycle arrest and cell apoptosis in colorectal cancer (CRC) cells. HCT116 and RKO cells were transduced with lentivirus encoding either anti-SNTB1 small hairpin RNA (sh-SNTB1) or control shRNA (sh-Ctrl). **a** Cell cycle progression in HCT116 (upper panel) and RKO (lower panel) cells was determined by PI staining and FACS analysis. Representative plots (left panel) and percentage of cells (right panel) at different stages (G0/G1, G2/M and S phases) are presented. *P < 0.05. **b** Apoptosis of HCT116 (upper panel) and RKO (lower panel) cells was determined by Annexin V staining and FACS analysis. Representative plots (left panel) and percentage of apoptotic cells (right panel) are presented. *P < 0.05. All experiments were performed in triplicate

QPCR and Western-blot analysis confirmed significant down-regulation of SNTB1 mRNA and protein levels in both HCT116 and RKO cells after transduction with sh-SNTB1 (Fig. 3a–c). More importantly, SNTB1 knockdown profoundly reduced the viability and colony formation of HCT116 and RKO cells (Fig. 3d, e). Moreover, SNTB1 knockdown markedly increased the percentage of cells in the G0/G1 phase, while decreased the percentage of cells in the S phase in HCT116 and RKO cells (Fig. 4a). Moreover, flow cytometry with annexin V staining demonstrated that SNTB1 knockdown remarkably increased the percentage of apoptotic cells (Fig. 4b).

### SNTB1 knockdown suppresses CRC tumor growth in vivo

**Fig. 5:**
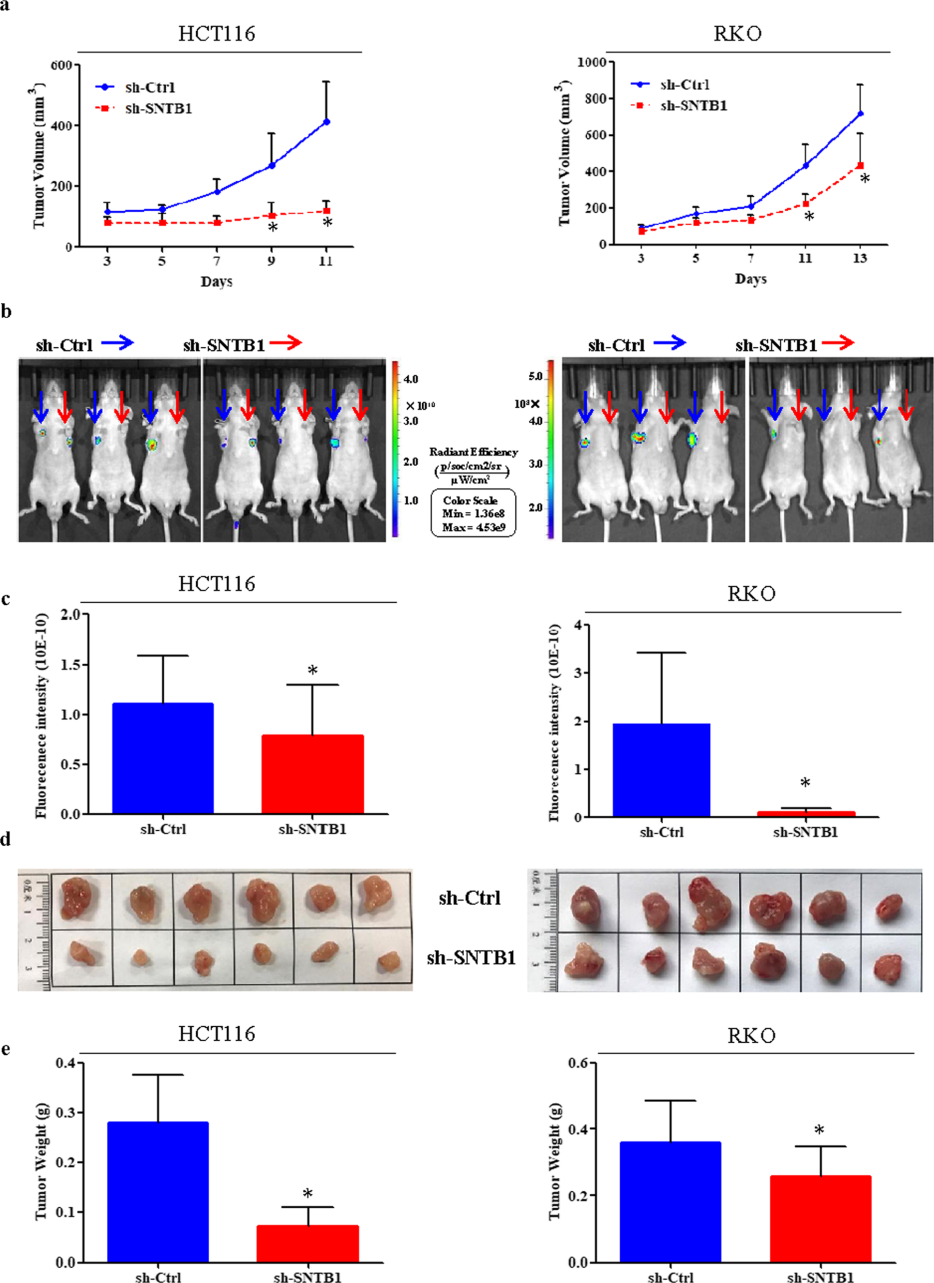
SNTB1 knockdown inhibits colorectal cancer (CRC) cell growth in vivo. A xenograft nude mouse model was established to investigate the tumor growth of HCT116 (left panel) and RKO (right panel) cells in vivo. A total of 1.0 × 10^6^ transduced cells were injected subcutaneously into the forelimb axillae (sh-Ctrl: left; sh-SNTB1: right) of BALB/c nude mice; these cells had been transduced with lentivirus encoding sh-Ctrl (left axilla) or sh-SNTB1 (right axilla). The tumor volumes (**a**), fluorescence of GFP (**b** and **c**), images of tumor and tumor weights (**d** and **e**) were determined and recorded. *P < 0.05

**Fig. 6:**
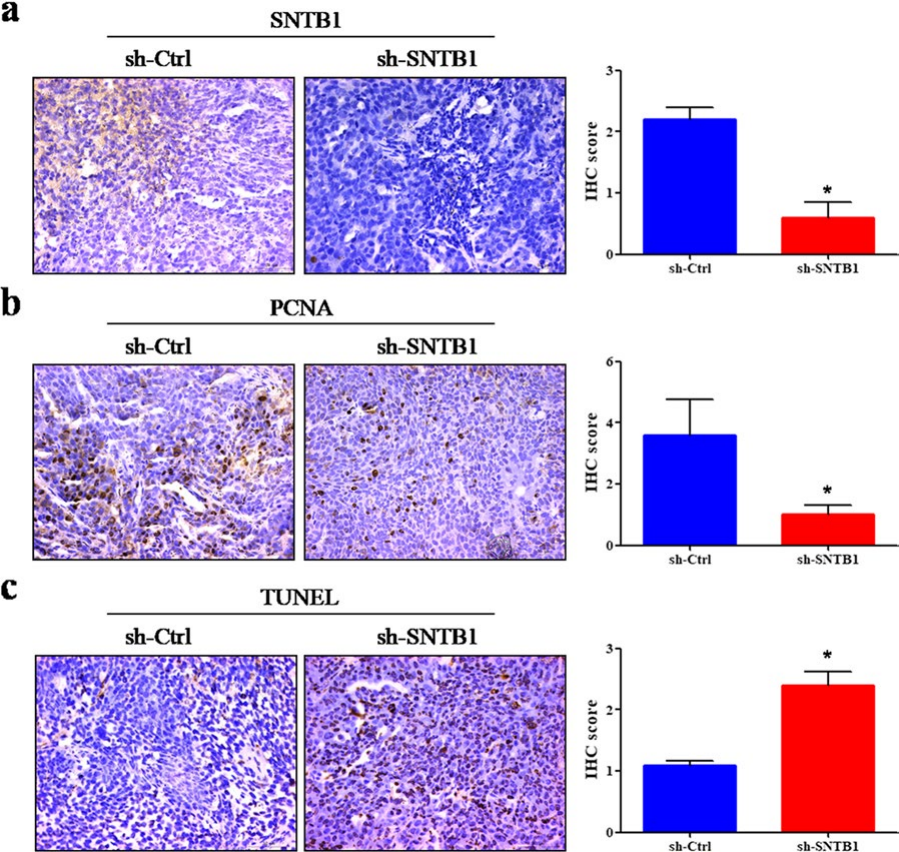
SNTB1 knockdown inhibits cell proliferation and induces cell apoptosis *in vivo*. IHC was performed to detect SNTB1 (**a**) and PCNA (**b**) expression in tumor sections, and TUNEL assay (**c**) was used to determine the apoptotic cells in tissues of both sh-Ctrl and sh-SNTB1. The representative images of IHC analysis or TUNEL assay were taken at a magnification of 400× (left panel) and IHC scores were calculated (right panel). *P < 0.05

We further determined the effects of SNTB1 knockdown on tumor growth in vivo. By evaluating tumor volume (Fig. 5a), intratumoral green fluorescent protein (GFP) fluorescence (Fig. 5b, c), tumor size (Fig. 5d), and tumor weight (Fig. 5e), we found that SNTB1 knockdown significantly suppressed tumor growth of both HCT116 and RKO cells in vivo. IHC analysis and TUNEL assay showed that SNTB1 knockdown (Fig. 6a) significantly decreased the expressions of proliferating cell nuclear antigen (PCNA) (Fig. 6b) and increased cell apoptosis in the HCT116 xenograft (Fig. 6c).

### SNTB1 knockdown up-regulates the expression of PKN2 and inhibits the phosphorylation of ERK and AKT

**Fig. 7:**
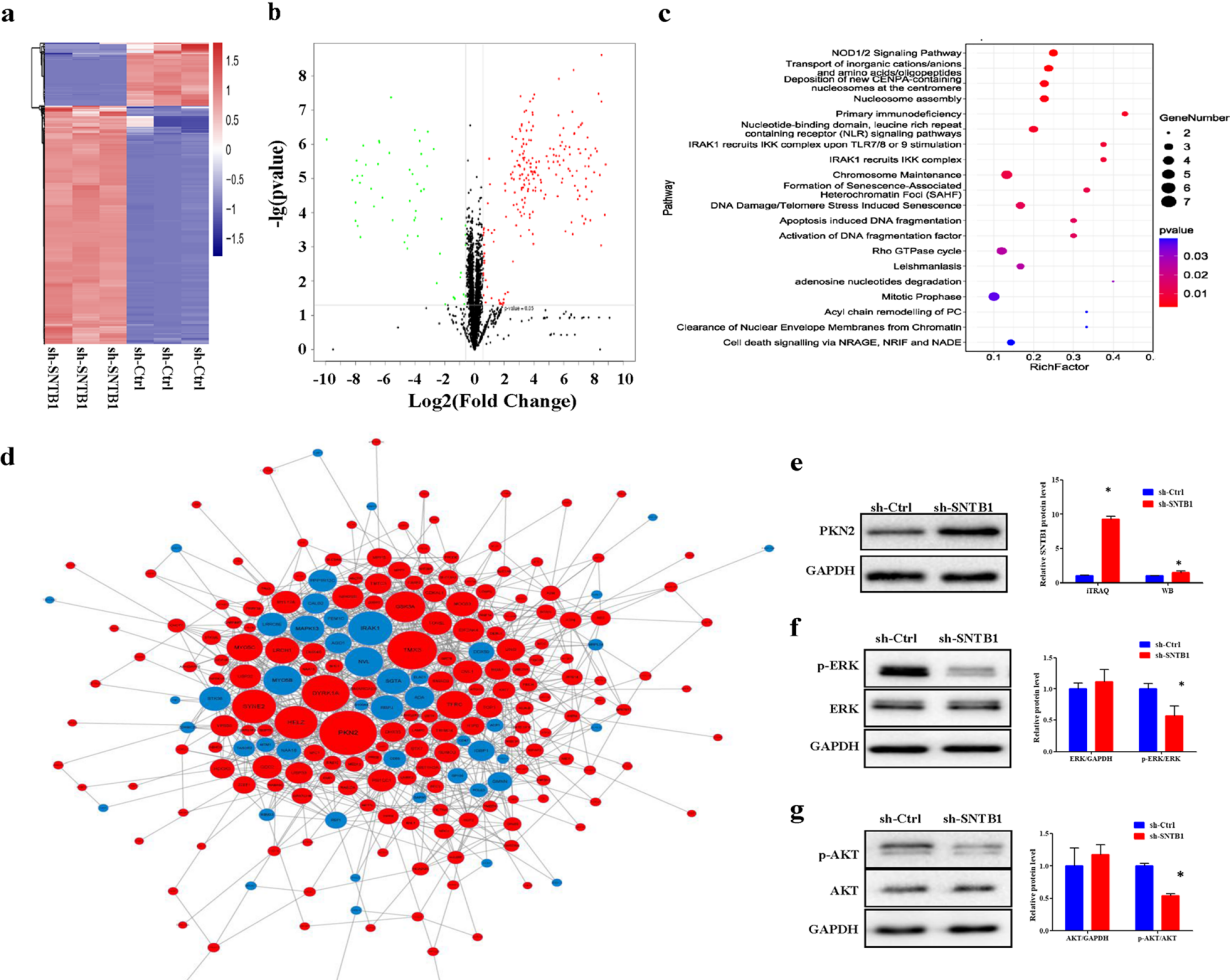
SNTB1 knockdown increases PKN2 protein expression and decreases p-ERK and p-AKT expression. HCT116 cells were transduced with lentivirus encoding either anti-SNTB1 shRNA (sh-SNTB1) or control shRNA (sh-Ctrl), and iTRAQ was used to identify DEPs. A hierarchical clustering plot (**a**) and a volcano plot (**b**) were used to identify DEPs (fold change ≥ 1.5, *P* < 0.05). **c** KEGG pathway enrichment analysis was performed to identify functionally related gene pathways. The top 20 enriched signaling pathways are shown. **d** Protein-protein interaction (PPI) network among DEPs was constructed. Red represent up-regulated proteins, while green represent down-regulated proteins. **e** The protein levels of PKN2 in HCT116 cells after SNTB1 knockdown were determined by Western-blot analysis. **f** The protein levels of p-ERK and ERK in HCT116 cells after SNTB1 knockdown were determined by Western-blot analysis. **g** The protein levels of p-AKT and AKT in HCT116 cells after SNTB1 knockdown were determined by Western-blot analysis. The representative images are shown in the upper panel and its expression was quantitated using ImageLab software in the lower panel. GAPDH was used as an internal control. The protein expression in sh-Ctrl was set as 1. The protein expression is presented as the fold change relative to the sh-Ctrl group *P < 0.05. All experiments were performed in triplicate

To further elucidate the underlying mechanism of SNTB1 knockdown on tumor growth suppression in CRC, iTRAQ methodology was applied to identify differentially expressed proteins (DEPs) in HCT116 cells after SNTB1 knockdown. As shown in Fig. 7, a total of 265 DEPs were identified, including 210 up-regulated and 55 down-regulated proteins (fold change ≥ 1.5, *P < *0.05) (all were listed in Additional file 9: Table S6 and Additional file 10: Table S7). Hierarchical clustering (Fig. 7a) and volcano (Fig. 7b) plots were used to identify DEPs. Enrichment analysis of the KEGG pathway showed these proteins to be enriched in multiple signaling pathways (Fig. 7c). A PPI network was constructed (Fig. 7d) and the top 10 hub genes were identified on the basis of their degree of connectivity in the PPI network: PKN2, TMX3, DYRK1A, IRAK1, SYNE2, HELZ, GSK3A, MYO5B, and NVL (Additional file 11: Table S8).

As a top hub protein, PKN2 expression was significantly up-regulated in HCT116 cells after SNTB1 knockdown. Therefore, we further explored the regulatory effects of SNTB1 knockdown on PKN2 expression and its downstream targets, including ERK and AKT pathways. Consistently, SNTB1 knockdown significantly increased the protein pression of PKN2 (Fig. 7e) and inhibited the phosphorylation of ERK (Fig. 7f) and AKT (Fig. 7 g). However, SNTB1 knockdown had no significant effects on the phosphorylation of c-jun or p38 MAPK in HCT116 cells (Additional file 3: Figure S3).

### Oncogenic activities of SNTB1 depend on PKN2

**Fig. 8:**
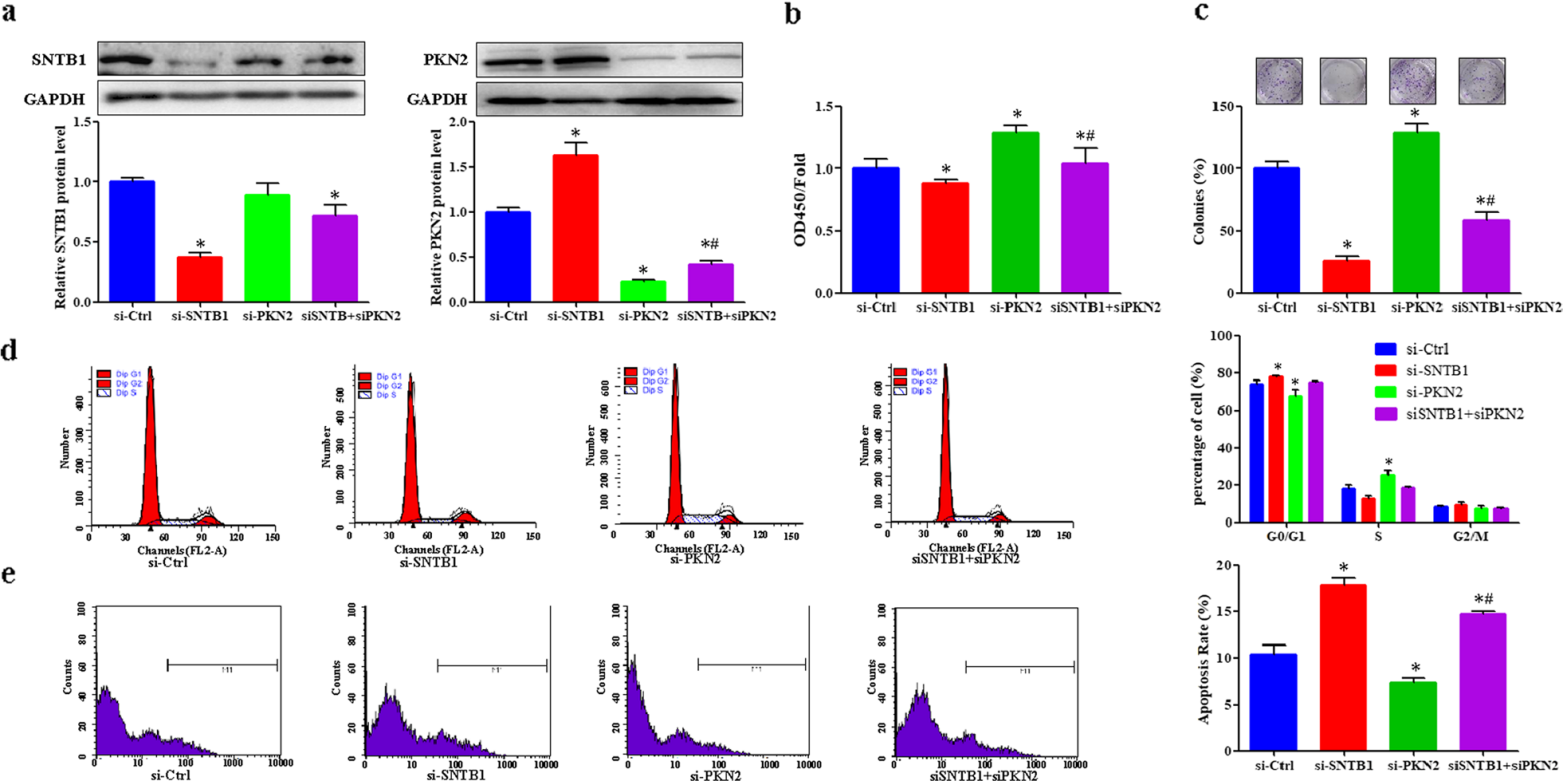
SNTB1 knockdown suppresses cell proliferation and induces cell apoptosis dependent on PKN2. HCT116 cells were transfected with si-SNTB1 or si-PKN2, or combination of si-SNTB1 and si-PKN2, or their related control siRNA (si-Ctrl). **a** The protein levels of SNTB1 (left) and PKN2 (right) in HCT116 cells were determined by Western-blot analysis. The representative images of PKN2, SNTB1 and GAPDH were shown and were quantitated using ImageLab software. GAPDH was used as an internal control and normalized to GAPDH. *P < 0.05, #*P* < 0.05 vs. si-PKN2. **b** The cell viability of CRC cells was determined by the CCK-8 assay. The cell viability in si-Ctrl was set as 1. Data were normalized to the viability of si-Ctrl and represented as the fold change. *P < 0.05 vs. si-Ctrl, #*P* < 0.05 vs. si-PKN2. **c** The colony formation assay was performed to determine cell survival. The images were taken and the number of colonies were calculated and normalized to the survival of control cells. *P < 0.05, #*P* < 0.05 vs. si-PKN2. **d** The cell cycle progression in HCT116 cells was determined by PI staining and FACS analysis. Representative plots (left panels) and percentage of cells (right panel) at different stages (G0/G1, G2/M and S phases) are presented. *P < 0.05. **e** Apoptosis of HCT116 cells was determined by Annexin V staining and FACS analysis. Representative plots (left panels) and percentage of apoptotic cells (right panel) are presented. *P < 0.05

Rescue experiments were performed to further explore the regulatory effect of SNTB1 on PKN2. Transfection of 3 different siRNA against PKN2 significantly decreased the expression of PKN2 and increased the cell viability in HCT116 cells (Additional file 2: Figure 2d–f). As each of the siRNA exhibited similar effects on PKN2 protein expression and cell viability, the si-PKN2-1 with higher cell viability was selected for the rescue experiment. Similar with sh-SNTB1 transduction, si-SNTB1 transfection significantly up-regulated PKN2 protein expression (Fig. 8a), decreased cell viability (Fig. 8b) and survival (Fig. 8c), as well as induced cell cycle arrest at G0/G1 phase (Fig. 8d), and cell apoptosis (Fig. 8e) in HCT116 cells, which were attenuated after PKN2 knockdown using si-PKN2 (Fig. 8a–e).

## Discussion

CRC is one of the most lethal cancers worldwide [1]. Although unhealthy dietary habits, environmental changes, and genetic aberrancies are contributors to the morbidity and mortality of this disease [23], the precise molecular mechanisms of CRC initiation and progression remain elusive. Therefore, the investigation into the molecular determines of CRC has the potential to lead to novel therapeutic targets, and diagnostic and prognostic biomarkers. Herein, we described the potential role of SNTB1 as an oncogene in CRC tissues.

As a modular adapter protein, SNTB1 binds and localizes various transmembrane and intracellular signaling molecules to the membrane [9]. Previous work identified SNTB1 over-expression as a candidate prognostic marker in patients with CRC [12]. Our current study identified the up-regulation of SNTB1 in CRC tissues at both mRNA and protein levels, suggesting that up-regulation of SNTB1 might be a common event during the development of CRC. Moreover, survival analysis based on cDNA array of CRC samples and online public databases indicate that there is a correlation between higher SNTB1 expression and shorter survival of CRC patients, thus confirming the potential clinical value of SNTB1 as a biomarker for disease prognosis.

Previous studies indicate that SNTB1 is involved in the initiation of autophagy in pancreatic cancer cells and has a protective effect on acute pancreatitis [7]. In addition, it has also been reported that SNTB1 is closely related to the occurrence of oral cancer and is considered to be a susceptibility locus for severe myopia [9, 10]. However, the functional roles of SNTB1 in CRC remain largely unknown. In order to further understand the function of SNTB1, we conducted a series of experiments and found that SNTB1 knockdown significantly suppressed CRC cell growth in vivo and in vitro by inhibiting cell viability and survival and inducing cell cycle progression and cell apoptosis. These findings suggest a potential oncogenic activity of endogenous SNTB1 in CRC. However, the effect of SNTB1 overexpression in CRC cell growth should be addressed in future studies.

The involvement of SNTB1 in cell proliferation and apoptosis is unknown, we therefore conducted iTRAQ analysis to identify DEPs in CRC cells after SNTB1 knockdown. iTRAQ analysis identified a total of 265 differentially expressed proteins (including 210 upregulated and 55 downregulated proteins). To identify the downstream targets of SNTB1, a PPI network was constructed. PPI analysis identified PKN2 as a hub protein in the PPI network, which was down-regulated in CRC cells after SNTB1 knockdown. Previous studies indicated that overexpression of PKN2 is involved in cell proliferation by regulating ERK/MAPK or AKT signaling pathways [24, 25] and inhibit M2 phenotype polarization of tumor-associated macrophages in CRC cells [26]. Our current study indicates that PKN2 knockdown significantly increases cell viability and cell survival, induces cell cycle arrest at G0/G1 phase and cell apoptosis, whereas at least partly reverses the SNTB1 knockdown medicated cell proliferation suppression, cell cycle arrest and cell apoptosis in CRC cells. These studies suggest that SNTB1 exhibits oncogenic activity at least partly dependent on PKN2.

Since abnormal activation of MAPK and AKT pathways contribute to multiple cellular processes, including cell survival, cell differentiation, apoptosis, invasion, and inflammation [27–30], we therefore assessed the regulatory effects of SNTB1 on PKN2 expression and those multiple down-stream signaling pathways. Interestingly, SNTB1 knockdown significantly decreased the levels of both p-ERK and p-AKT expression, while having no effect on p38 MAPK and p-JNK. However, the regulatory role of SNTB1 should be further addressed in future studies.

## Conclusions

**Fig. 9:**
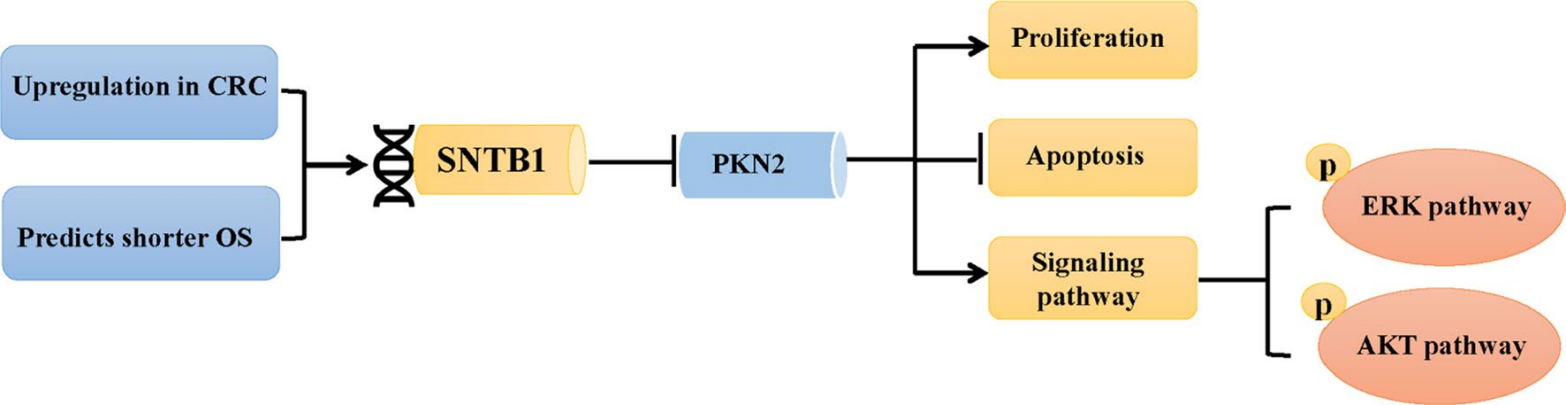
The summarize of SNTB1-ERK/AKT-CRC prognosis

In summary, our current study indicates that SNTB1 expression is upregulated in CRC tissues and correlates with CRC patient survival. In addition, we demonstrate SNTB1 has an essential role in CRC proliferation and apoptosis, which is mediated, in part, through the reduction of PKN2 expression and activation of EKR and AKT pathways (Fig. 9). Also, these studies highlight the potential of SNTB1 as a therapeutic target and biomarker for CRC.

## Supplementary Information

**Additional file 1: Figure S1.** Transduction of threeindependent SNTB1 specific shRNA lentivirus decreases the endogenous SNTB1 expressionin both mRNA and protein levels. HCT116 cells were transduced with one of three independent shRNAlentiviruses specific for sh-SNTB1 or sh-Ctrl.** a** SNTB1 mRNA expression in HCT116 cells was determined by qPCR. (**b** and **c**) SNTB1 protein expression in HCT116 cells was determined by western-blotanalysis. GAPDH was used as internal control. The integrated density of proteinband was assessed by ImageLab software. Protein expression is presented as thepercentage relative to the sh-Ctrl group (*P < 0.05). All experiments wereperformed in triplicate.

**Additional file 2: Figure S2.** Transfection of threeindependent SNTB1 or PKN2 specific siRNA decrease the endogenous SNTB1 or PKN2protein expression and their effects on cell viability. HCT116 cells were transfected with si-Ctrl or threeindependent siRNA for si-SNTB1 (**a**-**c**) or si-PKN2 (**d-f**).(**a**-**b**)SNTB1 or (**d-e**) PKN2 proteinexpression in HCT116 cells was determined by western-blot analysis. GAPDH wasused as internal control. The integrated density of protein band was assessedby ImageLab software. The protein expression in si-Ctrl was set as 1. The proteinexpression is presented as the fold change relative to the si-Ctrl group (*P< 0.05). (**c** and **f**) The cell viability of HCT116 cellsafter transfected with si-SNTB1 or PKN2 was determined by CCK8, the cell viability in si-Ctrl was set as 1.Data were normalized to the viability of sh-Ctrl and represented as the foldchange. *P < 0.05. All experiments were performed intriplicate.

**Additional file 3: Figure S3.** The proteinexpression of p-cJun, cJun, p-p38 MAPK, p38 MAPK in HCT116 cells after SNTB1knockdown. (**a** and **b**) Protein levelsof p-cJun, cJun, p-p38 MAPK andp38 MAPK in HCT116 cells after transduction withshRNA-SNTB1 or sh-Ctrl were determined by Western-blot analysis. GAPDH was usedas internal control. The integrated density of protein band was determinedusing ImageLab software. The protein expression in si-Ctrl was set as 1. The proteinexpression is presented as the fold change relative to the sh-Ctrl group (*P< 0.05). All experiments were performed in triplicate.

**Additional file 4: Table S1**. Oligonucleotidesequences for shRNA.

**Additional file 5: Table S2.** Primer sequences forQ-PCR.

**Additional file 6: Tables S3.** Clinic pathologicalfeatures of 79 CRC patients in cDNA array.

**Additional file 7: Table S4.** Clinic pathological features of 70 CRC patients in TMA.

**Additional file 8: Table S5.** Correlation between SNTB1 expression and clinicopathologicalcharacteristics.

**Additional file 9: Table S6.** The 55 down-regulated expressed proteins in iTRAQ methodology.

**Additional file 10: Table S7.** The 210 up-regulatedexpressed proteins in iTRAQ methodology.

**Additional file 11: Table S8.** The top 10 hub genes in the protein-protein interaction (PPI) network.

## Data Availability

The datasets generated and analyzed during the current study are available from the corresponding author on reasonable request.

## Abbreviations

CRC: Colorectal cancer
SNTB1: Syntrophin beta 1
qPCR: Quantitative real-time PCR
shRNA: Short hairpin RNAs
siRNA: Small interfering RNAs
DEGs: Differential expressed genes
TMA: Tissue microarray
iTRAQ: Isobaric tags for relative and absolute quantification
PKN2: Protein kinase N2
PPI: Protein–protein interaction
TUNEL: Terminal deoxynucleotidyl transferase dUTPnick end labeling
PCNA: Proliferating cell nuclear antigen
HCS: High-content screening
cDNA: Complementary DNA
PMSF: Phenylmethylsulfonyl fluoride
OD: Optical density
DEPs: Differential expressed proteins
MS: Mass spectrometry
2D-LC–MSMS: Two-dimensional liquid chromatography–mass spectrometry
GFP: Green fluorescent protein
