# Supplementary figures and images for: Upregulation of SNTB1 correlates with poor prognosis and promotes cell growth by negative regulating PKN2 in colorectal cancer
Source: Cancer Cell Int. 2021 Oct 18;21:547. doi: 10.1186/s12935-021-02246-7 (PMC8524951; doi:10.1186/s12935-021-02246-7)

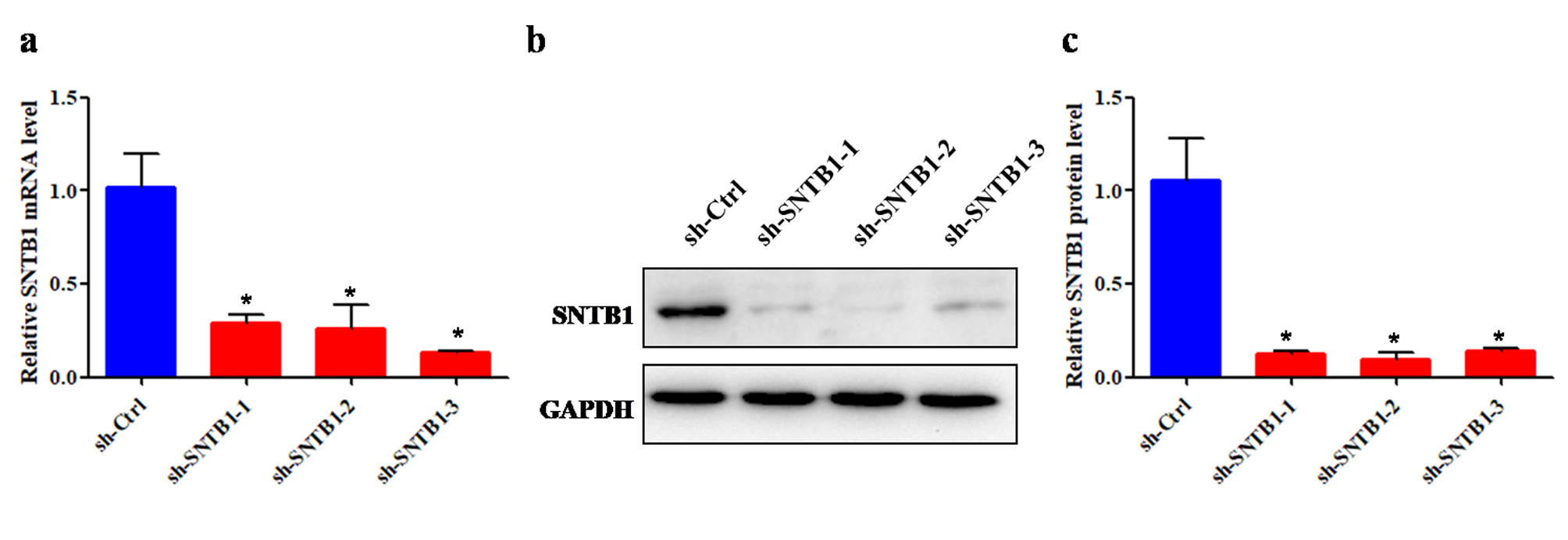

Supplement: Supplementary file 1 — Additional file 1: Figure S1. Transduction of threeindependent SNTB1 specific shRNA lentivirus decreases the endogenous SNTB1 expressionin both mRNA and protein levels. HCT116 cells were transduced with one of three independent shRNAlentiviruses specific for sh-SNTB1 or sh-Ctrl. a SNTB1 mRNA expression in HCT116 cells was determined by qPCR. (b and c) SNTB1 protein expression in HCT116 cells was determined by western-blotanalysis. GAPDH was used as internal control. The integrated density of proteinband was assessed by ImageLab software. Protein expression is presented as thepercentage relative to the sh-Ctrl group (*P < 0.05). All experiments wereperformed in triplicate. [file 12935_2021_2246_MOESM1_ESM.tif]

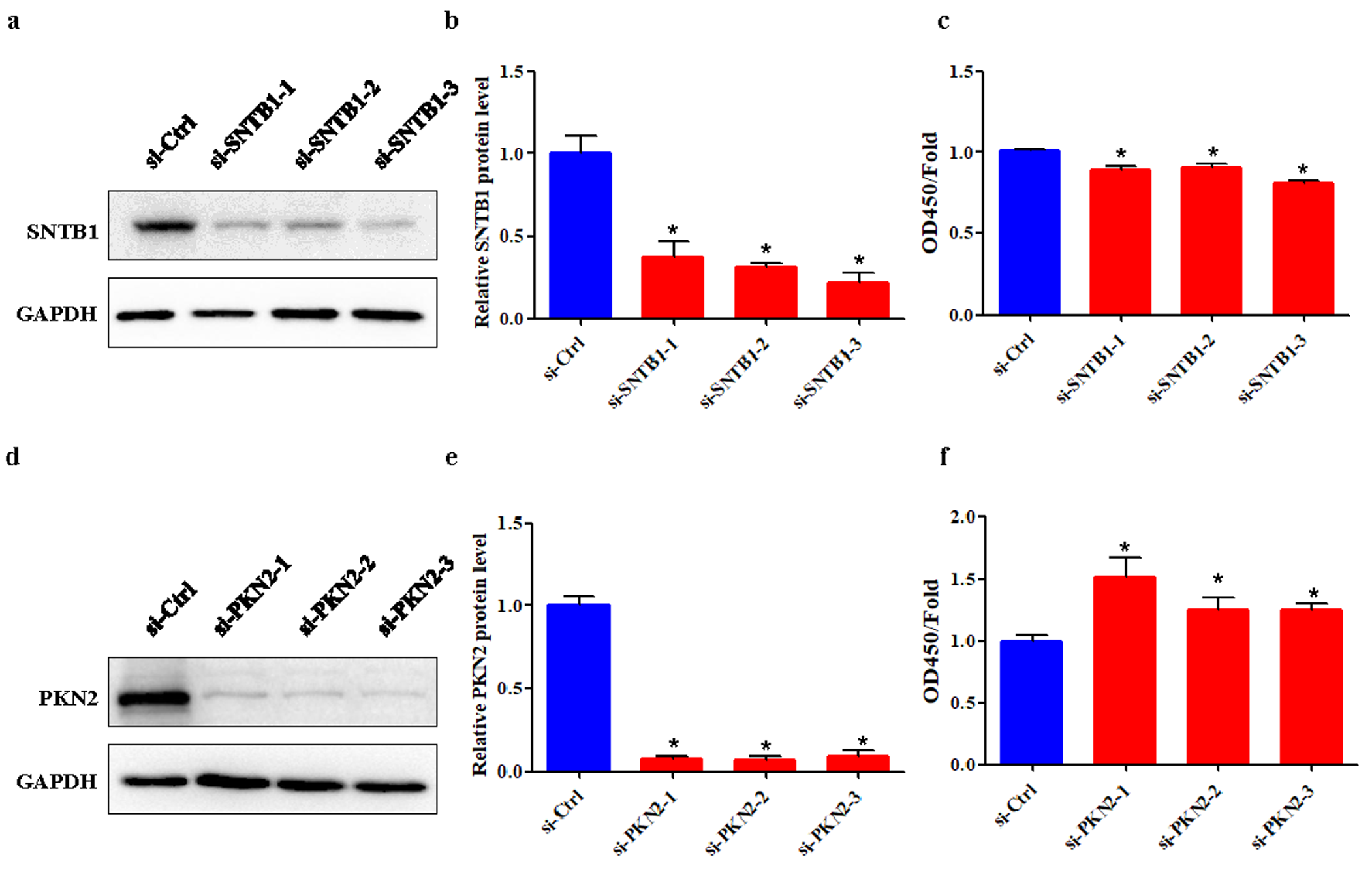

Supplement: Supplementary file 2 — Additional file 2: Figure S2. Transfection of threeindependent SNTB1 or PKN2 specific siRNA decrease the endogenous SNTB1 or PKN2protein expression and their effects on cell viability. HCT116 cells were transfected with si-Ctrl or threeindependent siRNA for si-SNTB1 (a-c) or si-PKN2 (d-f).(a-b)SNTB1 or (d-e) PKN2 proteinexpression in HCT116 cells was determined by western-blot analysis. GAPDH wasused as internal control. The integrated density of protein band was assessedby ImageLab software. The protein expression in si-Ctrl was set as 1. The proteinexpression is presented as the fold change relative to the si-Ctrl group (*P< 0.05). (c and f) The cell viability of HCT116 cellsafter transfected with si-SNTB1 or PKN2 was determined by CCK8, the cell viability in si-Ctrl was set as 1.Data were normalized to the viability of sh-Ctrl and represented as the foldchange. *P < 0.05. All experiments were performed intriplicate. [file 12935_2021_2246_MOESM2_ESM.tif]

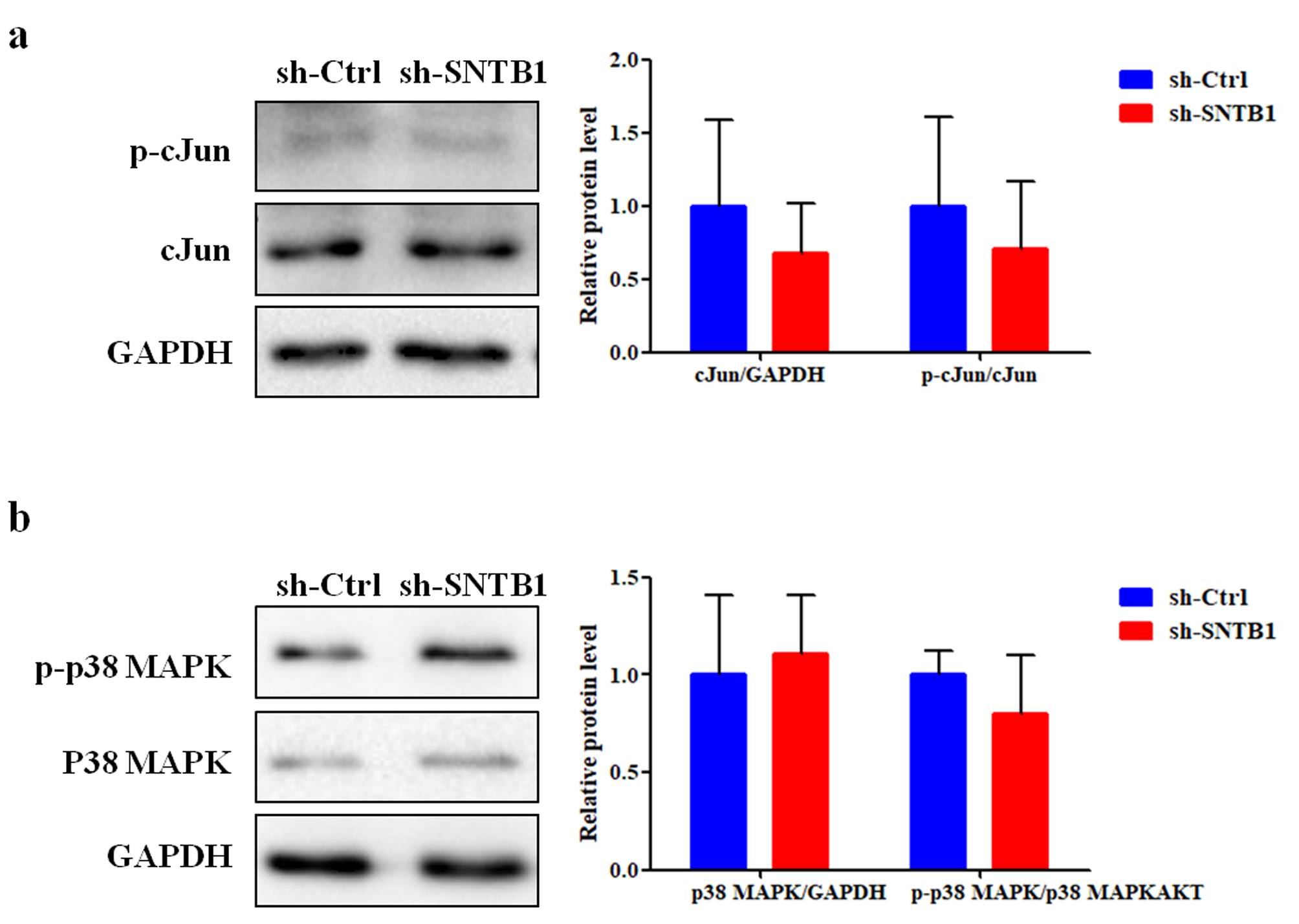

Supplement: Supplementary file 3 — Additional file 3: Figure S3. The proteinexpression of p-cJun, cJun, p-p38 MAPK, p38 MAPK in HCT116 cells after SNTB1knockdown. (a and b) Protein levelsof p-cJun, cJun, p-p38 MAPK andp38 MAPK in HCT116 cells after transduction withshRNA-SNTB1 or sh-Ctrl were determined by Western-blot analysis. GAPDH was usedas internal control. The integrated density of protein band was determinedusing ImageLab software. The protein expression in si-Ctrl was set as 1. The proteinexpression is presented as the fold change relative to the sh-Ctrl group (*P< 0.05). All experiments were performed in triplicate. [file 12935_2021_2246_MOESM3_ESM.tif]
