# Supplementary material for: Upregulation of SNTB1 correlates with poor prognosis and promotes cell growth by negative regulating PKN2 in colorectal cancer
Source: Cancer Cell Int. 2021 Oct 18;21:547. doi: 10.1186/s12935-021-02246-7 (PMC8524951; doi:10.1186/s12935-021-02246-7)
Supplement: Supplementary file 4 — Additional file 4: Table S1. Oligonucleotidesequences for shRNA. [file 12935_2021_2246_MOESM4_ESM.doc]

**Table S1. Oligonucleotide sequences for shRNA**

| Gene | Code | Sequence* |
| --- | --- | --- |
| SNTB1 | sh-SNTB1-1 | ccATACATTATGAGAATGGAT |
|  | sh-SNTB1-2 | cgCCCTATGTGAAGAAAGGAT |
|  | sh-SNTB1-3 | cgAGTCCATCTCGAACCAGAA |
| PLEKHG4 | sh-PLEKHG4-1 | GCAGACATTGGGTGTTTCCAT |
|  | sh-PLEKHG4-2 | GAGTACACTATGGAGAACTAT |
|  | sh-PLEKHG4-3 | CCGTCAACTATGTCCTGAAGT |
| JPH1 | sh-JPH1-1 | CAACGATTCATGCCCTGCTTT |
|  | sh-JPH1-2 | GCATTCTCAGTATCACGGCTA |
|  | sh-JPH1-3 | GCAAATTCAAGGACTGCACAT |
| CTPS1 | sh-CTPS1-1 | GCTCTCACATTACCTCCAGAA |
|  | sh-CTPS1-2 | CCCTTGTTGTTAGAGGAGCAA |
|  | sh-CTPS1-3 | TGATCTTGTAGCGGATGATTC |
| LRRC6 | sh-LRRC6-1 | GCCCAAGGTAGGAGAAGTAAT |
|  | sh-LRRC6-2 | CCTAAATGTGAATGAGCCCAA |
|  | sh-LRRC6-3 | CCTGTTTGTTTACTCCTGAAT |
| LY6G6F | sh-LY6G6F -1 | AGGAGCCGAAGACCAAGAATC |
|  | sh-LY6G6F -2 | ACGACGTCTTGGTGCTCAAAG |
|  | sh-LY6G6F -3 | TGTCCCTCACCACCTACTCTA |
| PLCB4 | sh-PLCB4-1 | CGCTGACATCAGATCACAAAT |
|  | sh-PLCB4-2 | GCAGGTTATATCAGGTCAATT |
|  | sh-PLCB4-3 | CGGGAAGTCTTCGGTAGAAAT |
| LRP8 | sh-LRP8-1 | CGAGTGGCATTAAGCCTTGAA |
|  | sh-LRP8-2 | CTCAAGAATGTCGTGGCACTA |
|  | sh-LRP8-3 | GACCTCAAGATTGGCTTTGAA |

*All oligonucleotides were double-stranded. In the case of shRNAs, only the 5'-3' strand is shown.
