# Supplementary material for: Upregulation of SNTB1 correlates with poor prognosis and promotes cell growth by negative regulating PKN2 in colorectal cancer
Source: Cancer Cell Int. 2021 Oct 18;21:547. doi: 10.1186/s12935-021-02246-7 (PMC8524951; doi:10.1186/s12935-021-02246-7)
Supplement: Supplementary file 5 — Additional file 5: Table S2. Primer sequences forQ-PCR. [file 12935_2021_2246_MOESM5_ESM.docx]

**Table S2. Primer sequences for Q-PCR**

| Gene | Primers (5`-3`) |
| --- | --- |
| SNTB1 | F: GGAGAGATTCAACTGGACCTTC  R: GCTGCTCACTACAGATGGTGTC |
| GAPDH | F: ATGGGGAAGGTGAAGGTCG  R: GGGGTCATTGATGGCAACAATA |

F, Forward；R, Reverse
