# Supplementary material for: Upregulation of SNTB1 correlates with poor prognosis and promotes cell growth by negative regulating PKN2 in colorectal cancer
Source: Cancer Cell Int. 2021 Oct 18;21:547. doi: 10.1186/s12935-021-02246-7 (PMC8524951; doi:10.1186/s12935-021-02246-7)
Supplement: Supplementary file 6 — Additional file 6: Tables S3. Clinic pathologicalfeatures of 79 CRC patients in cDNA array. [file 12935_2021_2246_MOESM6_ESM.docx]

**Table S3. Clinic pathological features of 79 CRC patients in cDNA array**

| Characteristic | | n (%) |
| --- | --- | --- |
| Age (years) | < 65 | 37 (46.8%) |
|  | ≥ 65 | 42 (53.2%) |
| Gender | Female | 33 (41.8%) |
|  | Male | 46 (58.2%) |
| Tumor location | Rectum | 0 (0%) |
|  | Colon | 79 (100%) |
| Clinical stage | Ⅰ | 2 (2.5%) |
|  | Ⅱ | 57 (72.2%) |
|  | Ⅲ | 19 (24.1%) |
|  | Ⅳ | 0 (0%) |
| T stage | T1 | 0 (0%) |
|  | T2 | 4 (5.1%) |
|  | T3 | 17 (21.5%) |
|  | T4 | 53 (67.1%) |
| N stage | N0 | 40 (50.6%) |
|  | N1 | 23 (29.1%) |
|  | N2 | 12 (15.2%) |
| M stage | M0 | 77 (97.5%) |
|  | M1 | 2 (2.5%) |
| Lymph node metastasis |  | 35 (44.3%) |
