# Supplementary material for: Upregulation of SNTB1 correlates with poor prognosis and promotes cell growth by negative regulating PKN2 in colorectal cancer
Source: Cancer Cell Int. 2021 Oct 18;21:547. doi: 10.1186/s12935-021-02246-7 (PMC8524951; doi:10.1186/s12935-021-02246-7)
Supplement: Supplementary file 7 — Additional file 7: Table S4. Clinic pathological features of 70 CRC patients in TMA. [file 12935_2021_2246_MOESM7_ESM.docx]

| **Table S4. Clinic pathological features of 70 CRC patients in TMA** | | |
| --- | --- | --- |
| Characteristic | | n (%) |
| Age (years) | < 65 | 25 (35.7%) |
|  | ≥ 65 | 45 (64.3%) |
| Gender | Female | 27 (38.6%) |
|  | Male | 43 (61.4%) |
| Tumor size | ≤ 5 cm | 33 (47.1%) |
|  | >5 cm | 37 (52.9%) |
| Clinical stage | Ⅰ | 0 (0%) |
|  | Ⅱ | 36 (51.4%) |
|  | Ⅲ | 34 (48.6%) |
|  | Ⅳ | 0 (0) |
| T stage | T1 | 1 (1.4%) |
|  | T2 | 3 (4.3%) |
|  | T3 | 55 (78.6%) |
|  | T4 | 9 (12.9%) |
| N stage | N0 | 49 (70.0%) |
|  | N1 | 17 (24.3%) |
|  | N2 | 4 (5.7%) |
| M stage | M0 | 68 (97.1%) |
|  | M1 | 2 (2.9%) |
| Lymph node metastasis |  | 21 (30.0%) |
| Distant metastasis |  | 2 (2.9%) |
