# Supplementary material for: Upregulation of SNTB1 correlates with poor prognosis and promotes cell growth by negative regulating PKN2 in colorectal cancer
Source: Cancer Cell Int. 2021 Oct 18;21:547. doi: 10.1186/s12935-021-02246-7 (PMC8524951; doi:10.1186/s12935-021-02246-7)
Supplement: Supplementary file 8 — Additional file 8: Table S5. Correlation between SNTB1 expression and clinicopathologicalcharacteristics. [file 12935_2021_2246_MOESM8_ESM.docx]

**Table S5. Correlation between SNTB1 expression and clinicopathological characteristics**

|  | Total (N=79) | SNTB1 mRNA expression | | 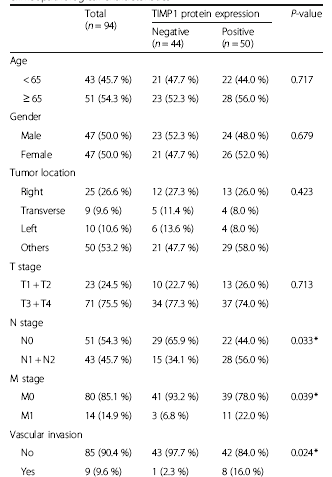P-value |
| --- | --- | --- | --- | --- |
|  |  | Low expression (n=15) | High expression (n=64) |  |
| Age |  |  |  |  |
| ≤ 65 | 41 (51.9%) | 10 (12.7%) | 31 (39.2%) | 0.208 |
| ＞ 65 | 38 (48.1%) | 5 (6.3%) | 33 (41.8%) |  |
| Gender |  |  |  |  |
| Male | 46 (58.2%) | 7 (8.9%) | 39 (49.4%) | 0.319 |
| Female | 33 (41.8%) | 8 (10.1%) | 25 (31.6%) |  |
| Pathology stage |  |  |  |  |
| Ⅰ | 2 (2.5%) | 0 (0%) | 2 (2.5%) | 0.661 |
| Ⅱ | 57 (72.2%) | 11 (13.9%) | 46 (58.2%) |  |
| Ⅲ | 19 (24.1%) | 4 (5.1%) | 15 (19.0%) |  |
| T stage |  |  |  |  |
| T1 | 0 (0%) | 0 (0%) | 0 (0%) | 0.598 |
| T2 | 4 (5.1%) | 1 (1.3%) | 3 (3.8%) |  |
| T3 | 17 (21.5%) | 2 (2.5%) | 15 (19.0%) |  |
| T4 | 53 (67.1%) | 12 (15.2%) | 41 (51.9%) |  |
| N stage |  |  |  |  |
| N0 | 40 (50.6%) | 9 (11.4%) | 31 (39.2%) | 0.192 |
| N1 | 23 (29.1%) | 6 (7.6%) | 17 (21.5%) |  |
| N2 | 12 (15.2%) | 0 (0%) | 12 (15.2%) |  |
| M stage |  |  |  |  |
| M0 | 77 (97.5%) | 15 (19.0%) | 62 (78.5%) | 0.494 |
| M1 | 2 (2.5%) | 0 (0%) | 2 (2.5%) |  |
