# Supplementary material for: Upregulation of SNTB1 correlates with poor prognosis and promotes cell growth by negative regulating PKN2 in colorectal cancer
Source: Cancer Cell Int. 2021 Oct 18;21:547. doi: 10.1186/s12935-021-02246-7 (PMC8524951; doi:10.1186/s12935-021-02246-7)
Supplement: Supplementary file 9 — Additional file 9: Table S6. The 55 down-regulated expressed proteins in iTRAQ methodology. [file 12935_2021_2246_MOESM9_ESM.docx]

**Table S6. The 55 down-regulated expressed proteins in iTRAQ methodology.**

| **Protein** | **Fold change** | **P value** | **Protein** | **Fold change** | **P value** | **Protein** | **Fold change** | **P value** |
| --- | --- | --- | --- | --- | --- | --- | --- | --- |
| **C18orf25** | **0.001025414** | **7.24666E-07** | **MRPL34** | **0.020528344** | **4.24442E-08** | **TJAP1** | **0.109897968** | **4.34257E-06** |
| **RBPJ** | **0.00337615** | **1.11546E-05** | **ZDHHC20** | **0.02209187** | **0.000165464** | **SGTA** | **0.114149389** | **4.29549E-07** |
| **NVL** | **0.00391855** | **0.000172415** | **KLHL26** | **0.038024744** | **0.000737164** | **OCIAD2** | **0.149718219** | **1.37918E-06** |
| **DDX59** | **0.003934272** | **5.00637E-05** | **BSDC1** | **0.043723381** | **0.001114436** | **PPP1R12C** | **0.172117484** | **0.011511268** |
| **NAA16** | **0.004097337** | **2.99033E-06** | **GMNN** | **0.044223131** | **0.000467574** | **YPEL5** | **0.199208709** | **0.001577836** |
| **AGO1** | **0.004271408** | **8.46058E-06** | **ABR** | **0.048509352** | **5.57629E-06** | **CD47** | **0.215360521** | **0.000151495** |
| **CHURC1** | **0.004345458** | **0.000245274** | **STK38** | **0.049125579** | **4.51685E-05** | **FEM1C** | **0.256441479** | **0.048080836** |
| **CXorf56** | **0.00479059** | **1.33243E-05** | **WBP4** | **0.055394159** | **7.41638E-06** | **GPR107** | **0.37856683** | **0.029629695** |
| **CCNY** | **0.004932967** | **0.000516815** | **MBOAT7** | **0.060153163** | **0.000165227** | **RSF1** | **0.38715385** | **0.030941812** |
| **GALNT3** | **0.005358602** | **8.31578E-06** | **ACP1** | **0.062217556** | **3.84423E-07** | **STARD10** | **0.400868595** | **0.033182469** |
| **PRCC** | **0.005611912** | **1.08283E-06** | **TASOR2** | **0.063413737** | **0.000140615** | **CD55** | **0.515377405** | **0.007786358** |
| **LRRC8E** | **0.00633599** | **3.94646E-06** | **KLHL11** | **0.064618266** | **8.87246E-05** | **MINPP1** | **0.531327171** | **0.005728988** |
| **POLE3** | **0.00806897** | **1.98987E-05** | **ADA** | **0.069149882** | **1.44121E-05** | **SAP30** | **0.597583607** | **0.024586597** |
| **AIDA** | **0.009209629** | **6.58616E-05** | **SH3BP5L** | **0.07133647** | **0.000483362** | **SP100** | **0.622625317** | **0.047425014** |
| **MTM1** | **0.011229508** | **3.76919E-06** | **SSU72** | **0.078306812** | **7.97183E-07** | **CALB2** | **0.659638653** | **0.000442847** |
| **IGBP1** | **0.011665385** | **8.02779E-07** | **TRPC4AP** | **0.082944592** | **2.32792E-05** | **ELAC1** | **0.659699966** | **0.028070365** |
| **IRAK1** | **0.011917872** | **8.75848E-07** | **MAPK13** | **0.083827596** | **1.91348E-06** | **S100A4** | **0.659975761** | **0.000237559** |
| **TOR1A** | **0.013668928** | **3.62305E-05** | **MYO5B** | **0.094481607** | **2.06256E-05** | **FAM114A1** | **0.662115132** | **0.000883695** |
| **RBM33** | **0.019052283** | **1.78684E-06** |  |  |  |  |  |  |
