# Supplementary material for: Upregulation of SNTB1 correlates with poor prognosis and promotes cell growth by negative regulating PKN2 in colorectal cancer
Source: Cancer Cell Int. 2021 Oct 18;21:547. doi: 10.1186/s12935-021-02246-7 (PMC8524951; doi:10.1186/s12935-021-02246-7)
Supplement: Supplementary file 10 — Additional file 10: Table S7. The 210 up-regulatedexpressed proteins in iTRAQ methodology. [file 12935_2021_2246_MOESM10_ESM.docx]

**Table S7. The 210 up-regulated expressed proteins in iTRAQ methodology.**

| **Protein** | **Fold change** | **P value** | **Protein** | **Fold change** | **P value** | **Protein** | **Fold change** | **P value** |
| --- | --- | --- | --- | --- | --- | --- | --- | --- |
| **HIST1H1E** | **450.7591742** | **3.87528E-06** | **PHLDB3** | **28.61243127** | **2.20918E-06** | **C18orf32** | **9.253508869** | **7.5078E-06** |
| **HLA-B** | **431.3079407** | **0.000116381** | **DNLZ** | **27.19895868** | **2.95495E-05** | **ARPP19** | **9.238476972** | **4.2036E-05** |
| **KAT7** | **370.9782526** | **2.46117E-09** | **MCRIP1** | **25.7459664** | **3.83349E-05** | **QRSL1** | **9.12059416** | **1.15996E-06** |
| **LRRC20** | **368.0358834** | **5.58739E-08** | **SYNE2** | **22.37736205** | **1.29402E-05** | **DHX35** | **9.043968649** | **2.61143E-07** |
| **SUMO3** | **362.1312784** | **0.00091144** | **KIFAP3** | **22.04618431** | **2.25488E-05** | **DVL1** | **8.976171318** | **2.8455E-07** |
| **MYL12A** | **342.0752741** | **7.89802E-06** | **SLC38A1** | **21.62845273** | **1.09618E-05** | **COX20** | **8.966177218** | **1.89697E-05** |
| **ARMC10** | **324.044425** | **3.33278E-08** | **GSK3A** | **20.09738925** | **3.18953E-06** | **IVNS1ABP** | **8.920838461** | **2.10824E-05** |
| **SLC9A6** | **299.7574485** | **1.60612E-06** | **LAMP2** | **19.32695101** | **7.96631E-06** | **ERMP1** | **8.863148501** | **0.00012478** |
| **G3BP2** | **279.5317065** | **1.31721E-06** | **LPCAT1** | **17.81795716** | **3.3123E-06** | **KNSTRN** | **8.604819723** | **0.000479896** |
| **FKBP15** | **264.3250405** | **6.57762E-06** | **CSRP2** | **17.73926312** | **4.78839E-06** | **PTPMT1** | **8.550318044** | **3.93681E-08** |
| **DERL1** | **234.1169144** | **2.39919E-05** | **NT5C3A** | **17.08987894** | **1.84094E-05** | **CEP85** | **8.485808299** | **4.09825E-07** |
| **C20orf27** | **225.8617648** | **2.0813E-06** | **IKBKG** | **17.04124517** | **2.29337E-05** | **MPP5** | **8.420611651** | **9.57246E-05** |
| **SERPINA7** | **220.4155182** | **0.000166657** | **FAM83D** | **16.95503582** | **6.73835E-06** | **DMAC2** | **8.33363279** | **8.02887E-06** |
| **NAA15** | **204.8462653** | **2.57992E-06** | **NAA30** | **16.59643619** | **6.28606E-06** | **ARHGEF18** | **8.310429113** | **4.41262E-06** |
| **PIP5K1A** | **203.6026448** | **4.40482E-06** | **MID1** | **16.12801528** | **3.51411E-08** | **BCS1L** | **8.195506818** | **3.57523E-07** |
| **KNL1** | **193.9167243** | **6.37745E-07** | **HIST1H2AJ** | **15.95987836** | **6.41125E-06** | **FDFT1** | **8.174768592** | **2.03574E-06** |
| **PDE6D** | **185.8444808** | **4.06337E-05** | **EEF1AKMT4** | **15.79856698** | **1.01653E-05** | **EIF2B5** | **8.157675272** | **1.02089E-05** |
| **CYBA** | **181.3799654** | **1.01199E-05** | **NCAPD3** | **15.6246659** | **0.000824884** | **RPP25L** | **8.03241175** | **4.02312E-06** |
| **CDC42BPG** | **179.1177726** | **1.74142E-05** | **PRPF39** | **15.36466855** | **2.22688E-05** | **CNOT10** | **7.73580179** | **4.02644E-06** |
| **GET4** | **175.87617** | **1.3983E-06** | **ARHGAP10** | **15.31355649** | **4.55096E-08** | **CHTOP** | **7.536717385** | **8.08349E-05** |
| **APOH** | **175.6668468** | **0.000153974** | **VGF** | **15.2980064** | **2.94289E-07** | **VPS50** | **7.329404015** | **3.04051E-06** |
| **MARCH5** | **173.0617902** | **4.36642E-06** | **CAMK1D** | **15.13364462** | **9.43983E-05** | **ACTR5** | **7.122584906** | **5.66674E-05** |
| **SLC1A5** | **164.1937378** | **0.000208597** | **TUBGCP4** | **15.05516138** | **0.000424241** | **NFIC** | **6.938211599** | **0.000235869** |
| **LPCAT3** | **150.5686555** | **0.000126185** | **ZCCHC3** | **15.03064859** | **1.35701E-07** | **STBD1** | **6.88566241** | **7.03175E-07** |
| **ABHD3** | **149.659559** | **2.42473E-07** | **RAB22A** | **14.97474546** | **7.50563E-06** | **GGA3** | **6.512449311** | **2.55479E-05** |
| **SMAD3** | **141.3159115** | **2.70508E-07** | **UNG** | **14.8127853** | **1.17179E-07** | **LONP2** | **6.510507349** | **4.43813E-06** |
| **ZNF346** | **136.7807668** | **1.82236E-06** | **NUF2** | **14.80740209** | **2.54794E-06** | **SORBS3** | **6.272885068** | **1.061E-05** |
| **ATP6** | **134.2167914** | **3.2363E-05** | **UHRF2** | **14.73641485** | **6.38234E-06** | **SRPRB** | **6.16321917** | **5.99979E-05** |
| **GPATCH8** | **128.0847629** | **3.1321E-07** | **TEAD1** | **14.10158617** | **3.89077E-07** | **MYADM** | **6.004002627** | **0.002693357** |
| **NISCH** | **127.8348091** | **1.38727E-06** | **HELZ** | **13.86637121** | **6.23106E-06** | **PTGR2** | **5.939429698** | **6.54053E-06** |
| **PKD1** | **123.5686514** | **4.02445E-05** | **LRRFIP2** | **13.84700489** | **7.64285E-07** | **EXOSC3** | **5.822504831** | **6.02538E-07** |
| **RAB12** | **122.835872** | **2.22822E-05** | **SRFBP1** | **13.6069812** | **3.62292E-06** | **GSTO2** | **5.783952693** | **1.58714E-06** |
| **DHPS** | **117.6007304** | **5.99151E-06** | **BET1L** | **13.60449454** | **3.68399E-06** | **ATP6V1F** | **5.637796996** | **9.89585E-06** |
| **MED13** | **107.9622392** | **1.62728E-05** | **CIAO3** | **13.55218727** | **4.73047E-06** | **ZNF428** | **5.473583143** | **7.52646E-06** |
| **CDC26** | **105.5010279** | **4.81186E-07** | **KHDRBS3** | **13.3528137** | **5.55696E-08** | **YIPF6** | **4.735370131** | **0.021224023** |
| **CAMSAP2** | **103.9845793** | **4.80164E-07** | **RFC1** | **13.14025173** | **0.000140787** | **TONSL** | **4.347306581** | **0.000131728** |
| **FAM177A1** | **103.1031231** | **4.94519E-07** | **DCP1A** | **13.10413841** | **0.000113112** | **PBXIP1** | **4.157924647** | **2.26632E-05** |
| **FAM122B** | **101.1815519** | **6.70019E-09** | **SLC4A2** | **12.7750473** | **1.42143E-05** | **EMC4** | **4.137420523** | **0.023565319** |
| **RAB40C** | **96.76990459** | **0.000425881** | **TRIO** | **12.66239197** | **0.000318728** | **EIF2AK4** | **4.117336349** | **1.40935E-05** |
| **DYRK1A** | **95.14562985** | **1.12473E-07** | **PRPF38B** | **12.64744306** | **7.52284E-07** | **TMTC3** | **3.884564393** | **0.034390855** |
| **ZZEF1** | **95.01478501** | **8.41462E-06** | **INTS4** | **12.57960965** | **2.26465E-07** | **TMEM43** | **3.867256771** | **0.043554534** |
| **RNASEH1** | **92.12925056** | **0.000191141** | **GINS3** | **12.51579156** | **2.95293E-06** | **CNOT7** | **3.816219064** | **0.043580042** |
| **ARFIP1** | **85.15209647** | **1.96646E-06** | **SIRT6** | **12.46457296** | **1.11449E-05** | **MYO5C** | **3.730959882** | **0.049813782** |
| **MYCBP2** | **85.08687353** | **6.0023E-06** | **ATG12** | **12.1990905** | **2.02964E-05** | **KDSR** | **3.475508879** | **0.046229203** |
| **NFKBIB** | **83.02010933** | **1.20061E-06** | **DHX40** | **12.18565459** | **7.87857E-06** | **SLC12A2** | **3.28242494** | **0.046901711** |
| **CDKAL1** | **79.64056852** | **5.22225E-06** | **GPR108** | **11.92903656** | **2.31562E-05** | **TMX3** | **3.272400308** | **0.043221846** |
| **GCC2** | **70.12788391** | **1.1433E-06** | **LRCH1** | **11.45991836** | **3.42431E-06** | **ARHGAP35** | **3.12136083** | **0.036529998** |
| **MYO19** | **69.44622371** | **0.000348099** | **DCP1B** | **11.3150111** | **3.98083E-07** | **HIST1H1C** | **2.517025132** | **0.00031941** |
| **MRPL53** | **65.89261007** | **1.40124E-06** | **TRIM24** | **11.17830694** | **9.91311E-07** | **TLCD1** | **2.447390444** | **0.000703116** |
| **CARNMT1** | **63.54117116** | **2.94476E-06** | **FERMT1** | **11.15161978** | **9.84913E-06** | **LEMD2** | **2.243937754** | **0.000392289** |
| **MKRN2** | **63.04907509** | **3.35217E-07** | **GMFB** | **11.0576633** | **1.02899E-07** | **HBA1** | **2.218114148** | **0.031831245** |
| **ZCCHC8** | **60.10411723** | **1.01388E-05** | **HOOK3** | **10.51657262** | **3.71861E-05** | **PHKA2** | **2.060377591** | **1.47004E-05** |
| **RPS14** | **59.14654327** | **8.62065E-07** | **HIST2H3PS2** | **10.34069044** | **5.09481E-05** | **DCTN4** | **1.973395506** | **2.6527E-06** |
| **REEP4** | **58.4936469** | **1.30471E-05** | **VPS37C** | **10.28591917** | **2.44252E-06** | **HIST2H4B** | **1.899864892** | **0.000320405** |
| **MED18** | **55.01521907** | **1.0929E-06** | **NPC1** | **10.26629348** | **1.56859E-05** | **H1F0** | **1.851398153** | **5.28576E-05** |
| **RB1CC1** | **53.37577827** | **5.4208E-07** | **ARHGEF10** | **10.21517407** | **1.12232E-07** | **ND2** | **1.813921222** | **0.014756911** |
| **SPAG7** | **52.71769564** | **4.00389E-06** | **PEX5** | **10.19156563** | **5.23668E-06** | **TFRC** | **1.680014599** | **0.009468101** |
| **MRPS17** | **51.57427283** | **2.17072E-06** | **PPCDC** | **10.17787087** | **5.49227E-06** | **NDUFB1** | **1.677511895** | **0.042217643** |
| **SMARCAD1** | **50.98429989** | **1.21376E-08** | **RBM47** | **10.13027842** | **0.000108413** | **FAM162A** | **1.586477209** | **0.00252989** |
| **STX7** | **49.91116526** | **1.42083E-07** | **ZBTB8OS** | **10.08554793** | **1.12242E-05** | **FAM3C** | **1.582833517** | **0.001552725** |
| **XPO4** | **48.1187916** | **3.19984E-06** | **USP33** | **10.06585108** | **1.48317E-05** | **EMD** | **1.575194654** | **0.002971292** |
| **C5orf51** | **47.88532488** | **3.32154E-05** | **USP32** | **10.06441007** | **1.59392E-06** | **PREB** | **1.570028123** | **0.000869717** |
| **NDC1** | **44.94045222** | **2.15283E-06** | **TMED9** | **9.975110796** | **2.86818E-06** | **FTL** | **1.562285945** | **0.000283786** |
| **CASP2** | **44.92692149** | **7.68875E-06** | **STK38L** | **9.830828735** | **9.70296E-07** | **TOP1** | **1.548570728** | **0.001870949** |
| **HJURP** | **40.08346999** | **2.03159E-06** | **HMGN1** | **9.64036024** | **7.12025E-07** | **SSR4** | **1.547664496** | **0.000381517** |
| **SVIL** | **39.51622169** | **1.3948E-06** | **SHC1** | **9.619717634** | **6.90145E-05** | **ERO1A** | **1.537122744** | **0.000309573** |
| **UBE2V1** | **39.38297748** | **3.28226E-06** | **FECH** | **9.580831974** | **2.51935E-06** | **SEPT5** | **1.531907001** | **0.025944547** |
| **MOCS3** | **33.57252243** | **1.31911E-06** | **NDUFAF1** | **9.563145787** | **1.8396E-05** | **ABCD3** | **1.512086303** | **0.006632851** |
| **MPP7** | **32.11304353** | **5.03906E-06** | **RNF14** | **9.538601344** | **1.87773E-06** | **RGP1** | **1.511647793** | **0.000455643** |
| **HS6ST2** | **30.73725965** | **3.19758E-06** | **PKN2** | **9.280553937** | **7.51832E-06** | **A2M** | **1.501969312** | **0.005923949** |
