# Supplementary material for: Upregulation of SNTB1 correlates with poor prognosis and promotes cell growth by negative regulating PKN2 in colorectal cancer
Source: Cancer Cell Int. 2021 Oct 18;21:547. doi: 10.1186/s12935-021-02246-7 (PMC8524951; doi:10.1186/s12935-021-02246-7)
Supplement: Supplementary file 11 — Additional file 11: Table S8. The top 10 hub genes in the protein-protein interaction (PPI) network. [file 12935_2021_2246_MOESM11_ESM.docx]

**Table S8: The top 10 hub genes in the protein-protein interaction (PPI) network.**

| Accession | Gene sample | Regulation | degree |
| --- | --- | --- | --- |
| NP_001307636.1 | PKN2 | up | 51 |
| NP_001337443.1 | TMX3 | up | 43 |
| NP_569122.1 | DYRK1A | up | 41 |
| NP_001020414.1 | IRAK1 | down | 37 |
| XP_005267516.1 | SYNE2 | up | 37 |
| XP_016880966.1 | HELZ | up | 36 |
| NP_063937.2 | GSK3A | up | 33 |
| NP_001073936.1 | MYO5B | down | 31 |
| XP_016856874.1 | NVL | down | 31 |
